# IsoPS-DIA: Dual Functionality of Absolute Targeted Quantification and Global Proteome Profiling

**DOI:** 10.1021/acs.analchem.5c05651

**Published:** 2026-01-27

**Authors:** Hsin-Ju Chan, Huan-Chi Chiu, Li-Yu Chen, Chi-Ting Lai, Chia-Yen Wang, Shr-Uen Lin, Sung-Liang Yu, Yu-Ju Chen

**Affiliations:** † Institute of Chemistry, 71552 Academia Sinica, Taipei 11529, Taiwan; ‡ Department of Chemistry, National Taiwan University, Taipei 10617, Taiwan; § Graduate Institute of Oncology, College of Medicine, National Taiwan University, Taipei 10051, Taiwan; ∥ Department of Clinical and Laboratory Sciences and Medical Biotechnology, 38005 National Taiwan ^∧^University College of Medicine, Taipei 100, Taiwan; ⊥ Department of Laboratory Medicine, National Taiwan University Hospital, Taipei 100, Taiwan

## Abstract

Current gene testing reveals only the mutation status yet lacks protein expression of the actual drug target. A comprehensive evaluation requires methods that integrate the absolute quantification of mutant proteins with global profiling of downstream signaling and resistance pathways. Here, we present an isotope pair-separated data-independent acquisition (IsoPS-DIA) strategy with a dual functionality of multiplexed absolute quantification and global proteome profiling in a single run. IsoPS-DIA features a dual-window design: narrow consecutive windows separate light/heavy-isotope-labeled peptide pairs to reduce coisolation interference and maximize usable fragment ions, while wide variable windows capture proteome-wide information. Using *EGFR* mutations (L858R, G719A, Del19) in lung cancer cell lines as a model, IsoPS-DIA achieved subfemtomole sensitivity (LOQ 36–222 amol), excellent linearity across 4 orders of magnitude (*R* ^2^ = 0.998–0.999), and high reproducibility (median CV ∼ 3%). For the first time, the method quantified endogenous *EGFR* and *KRAS* driver mutations alongside their wild-type counterparts, revealing allele-specific expression heterogeneity not captured by genomic variant allele frequency (VAFs). Simultaneously, IsoPS-DIA achieved >6,000 protein coverage across six cell lines, uncovering variability in *EGFR* signaling cascades and actionable variants such as *KRAS-G12S*. Benchmarking against parallel reaction monitoring (PRM), fixed scanning window (Fix-DIA) and variable scanning windows DIA (Var-DIA) confirmed IsoPS-DIA’s superior accuracy and reproducibility without compromising proteome coverage. IsoPS-DIA is compatible with both Orbitrap and quadrupole time-of-flight mass spectrometry (Q-TOF) platforms using standard software, and we provide an open-source window-design tool to facilitate adoption. These results demonstrate the unique capability of IsoPS-DIA to bridge genotype and proteotype through precise, scalable, and reproducible quantification, offering a broadly applicable platform for precision oncology and other applications.

## Graphical abstract

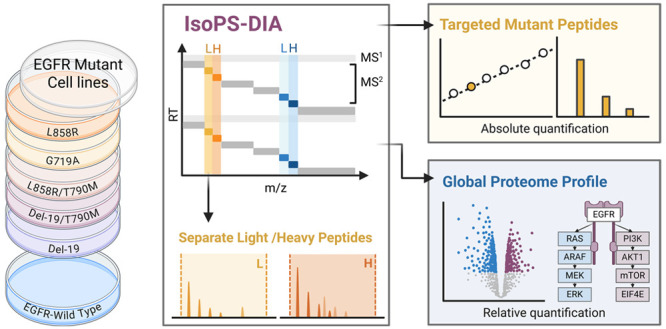

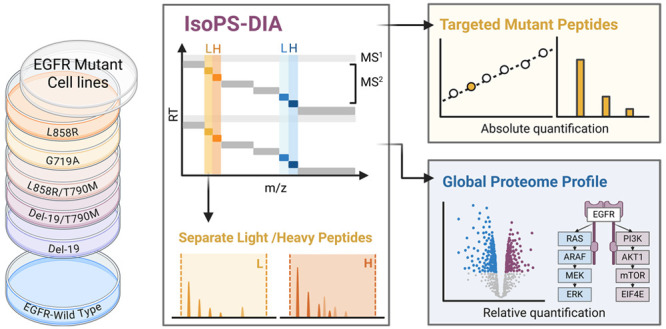

## Introduction

Genomic alterations drive disease initiation and progression, and many targeted therapies have been developed to target these actionable mutations. The U.S. Food and Drug Administration (FDA) has approved numerous small-molecule inhibitors and biologics for precision treatment. For example, *EGFR* mutations (L858R and Del19), present in 40–50% of Asian lung cancers, are widely used therapeutic targets,^1^ while *KRAS* G12C mutations, found in ∼30% of Caucasian cases, have recently led to inhibitor approval.^2^ These advances highlight how genomic testing has transformed oncology by guiding targeted therapy.^3,4^ Beyond cancer, SARS-CoV-2 protein variants also influence immune escape and vaccine effectiveness,^5^ demonstrating the broader impact of protein mutation-specific biology. However, DNA-based testing remains the standard for treatment decisions, which cannot determine whether mutant proteins are expressed at levels sufficient for therapeutic intervention.^6^ Because the drug response depends on both mutation status and protein abundance, there is a pressing need for assays that directly quantify druggable mutant proteins. Such protein-level measurements would complement genomic testing and strengthen the molecular basis for treatment decisions.

Data-independent acquisition (DIA) has recently emerged as a powerful proteomics strategy, offering higher reproducibility, fewer missing values compared with data-dependent acquisition (DDA), and greater multiplexing capacity than targeted MS. Since the early conceptualization^7,8^ and development of Sequential Window Acquisition of All Theoretical Mass Spectra (SWATH-MS),^9^ DIA has advanced substantially through innovations in acquisition schemes and hardware, leading to improved proteome coverage.^10–12^ Recent efforts have also integrated DIA with targeted MS modes for achieving both targeted protein detection and global profiling. For example, Martínez-Val et al. introduced Hybrid-DIA, which designed isotopically labeled peptides triggering PRM and DIA scans in orbitrap enabled by application programming interface (API),^13,14^ while Sanner et al. reported a mixed DIA–PRM workflow that alternates between DIA and PRM.^15^ Both methods enhance target sensitivity and quantitative performance, while retaining global profiling capacity.

Despite these advances, absolute protein quantification by DIA remains limited. Stable isotope-labeled peptides with calibration curves remain the gold standard,^16^ and several studies have attempted to integrate them into DIA workflows. Liu et al. quantified 41 plasma glycoproteins using SWATH-MS with isotope dilution.^17^ Kim et al. applied DIA for relative quantification of *RAS* mutations in biopsy samples.^18^ Husson et al. recently introduced Top3-ID-DIA for host cell protein quantification with performance comparable to SRM.^19^ However, under conventional DIA, coisolation of light and heavy peptide pairs generates overlapping fragment ions, complicating spectral deconvolution and reducing quantification performance. The restricted number of unique fragments further constrains quantification precision, particularly for low-abundance proteins and for distinguishing mutants from wild-type peptides.

Proteogenomics integrates genomic data into reference databases to enable detection of protein variants,^20,21^ yet distinguishing mutant from wild-type peptides remains challenging in shotgun proteomics due to high sequence similarity, low mutant abundance, and the complexity of peptide mixtures. Targeted MS methods such as selected/multiple reaction monitoring (SRM/MRM) and PRM provide the current gold standard for absolute quantification of predefined numbers of analytes with high specificity and sensitivity.^22,23^ Only a few studies have applied these approaches to protein variants. Tan et al. used SRM to validate single amino acid variant peptides identified from multisearch engines.^24^ Chen et al. quantified *BRAF* V600E mutants using an MRM assay.^25^ Despite their sensitivity, targeted MS methods are limited by a low multiplexing capacity and lack of global proteome coverage.

In this work, we introduce an Isotope Pairs-Separated DIA (IsoPS-DIA) strategy with dual functionality for simultaneous absolute targeted protein quantification and global proteomic profiling. Unlike conventional DIA, IsoPS-DIA employs dual scanning windows: (i) consecutive narrow windows that resolve light/heavy isotopic peptide pairs for accurate quantification and (ii) wide windows that enable global proteome profiling. Using lung cancer as a model, IsoPS-DIA quantified endogenous levels and activation pathways of clinically relevant driver mutations, including *EGFR* (L858R, Del19, G719A) and *KRAS* (G12S). To maximize the detectability across mutation sites, we further incorporated a multiple-protease digestion strategy. Benchmarking against conventional DIA with fixed scanning window (Fix-DIA), variable Q1 isolation windows DIA (Var-DIA)^26^ and PRM-MS demonstrated the superior ability of IsoPS-DIA to quantify protein variants, their wild-type counterparts, and signaling pathways linked to EGFR therapy. Notably, this is a generic method compatible with both the Orbitrap and Q-TOF platforms without the need for specialized software. To the best of our knowledge, IsoPS-DIA represents the first DIA-MS approach capable of absolute quantification of endogenous protein variants. Finally, we provide an open-source window-design tool to facilitate adoption of DIA design.

## Experimental Section

### Materials and Reagents

Triethylammonium bicarbonate buffer (TEABC), tris­(2-carboxyethyl) phosphine (TCEP), urea, and S-methylmethanethiosulfonate (MMTS) were purchased from Sigma-Aldrich (St. Louis, MO, USA). Lysyl Endopeptidase (LysC, mass spectrometry grade) was purchased from Wako Pure Chemical Industries, Ltd. (Osaka, Japan). Trypsin (modified, sequence grade) was purchased from Promega (Madison, WI, USA). Endoproteinase Glu-C (sequencing grade) was purchased from Roche (Basel, Switzerland). The BCA protein assay kit was obtained from Pierce (Rockford, IL, USA). C18 ZipTip Pipette Tips were purchased from Merck Millipore (Burlington, USA). EGF Receptor internal standard peptides were chemically synthesized with or without C-terminal lysine-(^13^C_6_^15^N_2_) and glutamic acid-(^13^C_5_^15^N_1_) to a 95% isotopic enrichment by Mission Biotech (Taipei, Taiwan).

### Cell Culture and Sample Preparation

The human lung adenocarcinoma cell lines A549 (*EGFR* wild-type), PC9 (*EGFR* Del19), and H1975 (*EGFR*-L858R/T790M) were purchased from ATCC (Virginia, USA) and grown in RPMI-1640 medium. The H3255 (*EGFR*-L858R), CL97 (*EGFR*-G719A/T790M), and CL68 (*EGFR* Del19/T790M) cell lines were kindly provided by Prof. Sung Liang Yu (The Department of Clinical Laboratory Sciences and Medical Biotechnology, National Taiwan University, Taiwan) and grown in RPMI-1640 medium. RPMI-1640 medium was supplemented with 0.375% (w/v) HEPES, 0.22% (w/v) sodium bicarbonate, 0.01% (w/v) sodium pyruvate, 10% (v/v) FBS, and 1% (v/v) penicillin–streptomycin–amphotericin solution at 37 °C with 5% CO_2_. The NSCLC cells were subjected to membrane protein extraction followed by gel-assisted digestion based on our previously reported protocol.^27^ To construct the calibration curve for absolute quantification of mutant and wild-type peptides, light synthetic peptides were pooled with serial dilutions (0.5, 1, 5, 25, 100, 500 fmol/μL), while heavy synthetic peptides were pooled with fixed concentration (25 fmol/μL). Furthermore, digested peptides from mouse lung tissues were also added to each calibrator to mimic the complex background in the samples and used in DIA-methods comparison.

### LC-MS/MS Analysis

LC-MS/MS analyses were performed on an Orbitrap Fusion Lumos Tribrid or an Orbitrap Eclipse Tribrid mass spectrometer coupled to an Ultimate 3000 RSLCnano system (Thermo Fisher Scientific, Bremen, Germany). To ensure consistent retention times, all digests were spiked with iRT peptides (Biognosys AG, Schlieren, Switzerland) and separated on a C18 column (Waters, CSH, 130 Å, 1.7 μm, 75 μm × 250 mm) at 300 nL/min. The mobile phases were (A) 0.1% formic acid in water and (B) 0.1% formic acid in acetonitrile (ACN). The 60 min gradient was as follows: 1–10% B in 3 min, 10–15% in 37 min, 15–25% in 8 min, 25–45% in 5 min, ramp to 90% in 2 min, and wash at 90% for 4 min. Common MS1 settings were 390–1250 *m*/*z*, 120,000 resolution, AGC target 4e5, and maximum injection time (IT) 50 ms. HCD collision energy was set at 25%. The MS1 windows in the range of 400–1000 *m*/*z* for the three DIA methods are as follows: **IsoPS-DIA**: 40 variable isolation windows (4–60 Th); **Fix-DIA**: 40 fixed windows of 15 Th; and **Var-DIA**: variable window sizes (9–63 Th) determined by MS1 ion intensity using swathTUNER.^26^ Full acquisition window details are listed in Supporting Table S1. MS2 scans were acquired at 15,000 resolutions with AGC 4e5 and IT 22 ms. **PRM**: Targeted isolation windows of 1.6 Th are set for precursor ions. Scheduled MS2 scans were acquired at 50,000 resolution, AGC 5e4, and IT 86 ms. An additional MS1 scan was collected at 30,000 resolution, AGC 4e5, and IT 54 ms.

The LC-MS settings of IsoPS-DIA for the Q-TOF analyzer are listed in Supporting Method 1.

### Spectral Library Construction and DIA Data Processing

Protein identification was achieved against the human reference proteome from UniProtKB/Swiss-Prot (*Homo sapiens*, release 2020.05; 20,295 entries). Full-length sequences of EGFR mutants (G719A, Del19, and L858R) and KRAS-G12S were appended to construct a custom mutation database. Mutant peptide spectral libraries were generated from DDA data acquired on 38 pooled samples of light- and heavy-labeled peptides spiked into PC9 membrane digests, searched in Proteome Discoverer version 2.5 (Thermo Fisher Scientific, Bremen, Germany). DIA raw files were processed in Spectronaut v19.0 (Biognosys AG, Schlieren, Switzerland) using the direct DIA+ workflow with default settings unless specified. The mouse tissue samples were searched against UniProtKB/Swiss-Prot (*Mus musculus*, release 2021.04; 17,074 entries) with the following parameters: Trypsin/P as the protease, peptide length of 7–52 residues, ≤2 missed cleavages, methylthio (C) as a static modification, and variable modifications of protein N-terminal acetylation, methionine oxidation, and asparagine/glutamine deamidation (≤3 per peptide). False discovery rates were controlled at 1% for precursors and proteins (experiment-wise) and 1% for proteins (run-wise). The NSCLC cell line samples were searched against human reference proteomes and used Lys-C/P as the protease; other parameters are the same as above.

### Absolute Protein Quantification

The PRM and DIA raw files were imported into Skyline^28^ and searched against mutation-specific spectral libraries. Absolute peptide abundances were determined from light-to-heavy (L/H) peak area ratios of the extracted ion chromatograms. Calibration curves were constructed for each peptide using linear regression (*y* = *mx* + *b*), with performance assessed by coefficient of determination (*R* ^2^) and residual standard deviation (Sy|x). Limits of detection (LOD) and quantification (LOQ) were calculated as LOD = 3Sa/b and LOQ = 10Sa/b, where Sa is the standard deviation of blanks and b is the regression slope. For calibration curves, the regression model was assessed for linearity and reliability using *R* ^2^ and Sy|x values. Calculation of the relative error follows the equation Relative Error = (Actual Value – Measured Value)/Actual Value.

### Statistical Analysis

Peptide spectral similarity between endogenous, library, and isotopic spectra was assessed in Skyline using cosine similarity scores based on the dot product of the precursor and fragment relative intensities. Signal-to-noise ratios and coefficients of variation across Fix-DIA, Var-DIA, IsoPS-DIA, and PRM were compared using Wilcoxon signed-rank tests in R. Reproducibility of relative quantification among the DIA methods was evaluated by Spearman correlation.

For global proteomic comparison across six lung cancer cell lines, protein intensities were log_2_-transformed, median-centered, and missing values were imputed per cell line with MissForest.^29^ Differential expression across cell lines was tested using Kruskal–Wallis with Benjamini–Hochberg FDR correction (adjusted *p* < 0.05). Quantitative comparison between cell lines was performed by using Wilcoxon rank-sum with FDR correction; proteins with ≥2-fold change and adjusted *p* ≤ 0.05 were considered significantly upregulated.

### Bioinformatic Analysis

Pathway enrichment analysis was conducted on significantly differentially expressed proteins using the STRING database^30^ by UniProt IDs, and enrichment was performed based on the Reactome Pathway Database^31^ using the *Homo sapiens* gene set as the background universe. Reactome pathways with adjusted *p*-values <0.05 were considered enriched. The top enriched pathways per cell line were visualized to facilitate functional interpretation.

### Determination of EGFR and KRAS Variant Allele Frequencies in Isogenic Cell Lines

EGFR and KRAS variant allele frequencies (VAFs) were obtained either from the Cell Model Passports database^32^ (for CL68 cells) or determined experimentally using the MassARRAY system^33^ (for H3255, H1975, CL97, PC9, and A549 cells). For sample preparation, genomic DNA was extracted using QIAamp kits and subjected to single nucleotide extension. Analysis was performed on a Bruker Autoflex MALDI-TOF MS platform. VAF was calculated as the mutant peak height relative to the total peak height. The method was validated for precision, accuracy, and sensitivity following TFDA-LDTS guidelines using FFPE, PBMC, and plasmid samples. The laboratory maintains external accreditation through CAP and EMQN proficiency testing.^34^

## Results and Discussion

### Design of IsoPS-DIA Strategy

In conventional DIA, fixed wide acquisition windows (10–20 Th) pose two major challenges for absolute protein quantification. First, coisolation of light and heavy peptides within the same window produces nearly identical fragment-ion patterns, leaving few unique fragment ions for quantification. For example, when using ^13^C_6_^15^N_2_-lysine labeling at the C-terminus, all *b*-ions of the light/heavy pair are identical (Figure 1A), restricting quantifiable ions for mutant peptides. Second, wide windows allow coelution of abundant nontarget peptides, causing ion suppression, lower signal-to-noise ratios, and reduced accuracy. To address these issues, IsoPS-DIA employs a dual-function acquisition design. (i) For absolute targeted quantitation, narrow 4 Th windows separate isotopic pairs of targeted peptides into two adjacent windows (Figure 1B), enabling independent full fragment-ion series and reducing ion suppression. The calibration curve was constructed by using different amounts of isotopic pairs of light and heavy peptides. (ii) For global proteomic profiling, wide variable windows (up to 60 Th) maximize proteome coverage and provide flexibility to cover target-specific pathways. This hybrid design achieves both enhanced accuracy of absolute protein quantification and comprehensive proteome profiling in single LC-MS/MS run (Figure 1C). To obtain the absolute quantification of targeted peptides, heavy-isotope internal standards are spiked into each sample. The light-to-heavy ratios were obtained by extracted ion chromatograms of fragment ions and fitted to external calibration curves to derive peptide concentrations by linear interpolation. For global proteome profiling, relative protein abundances are obtained from Spectronaut, which quantifies peptides using fragment-ion chromatograms and selects the top three abundant peptides to derive protein-level abundance.

**Figure 1:**
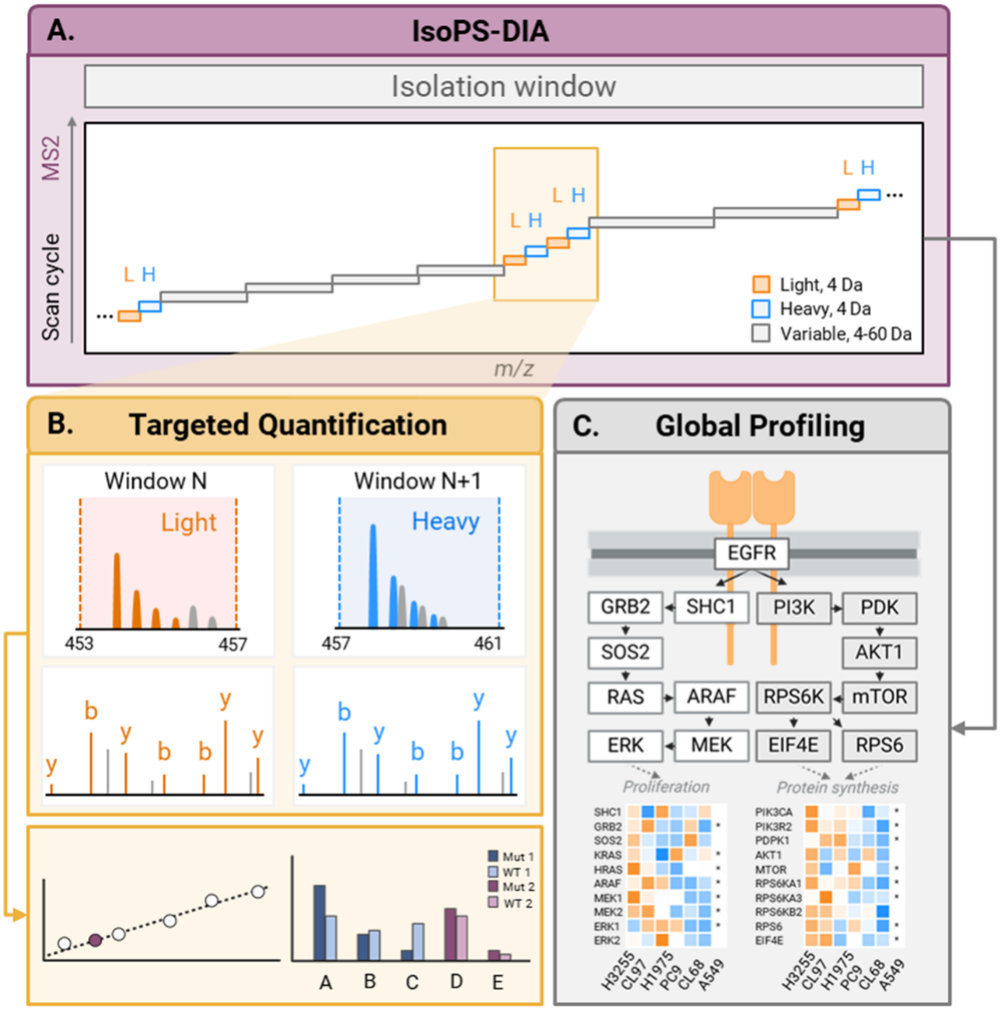
Schematic of IsoPS-DIA: (A) Schematic illustration of MS2 isolation window design of our IsoPS-DIA method, labeled with EGFR mutant and wild-type precursors. In IsoPS-DIA, 4 *m*/*z*-wide isolation windows were used for targeting EGFR precursors and separating the isotope pairs, along with other variable windows for untargeted protein identification. (B) The narrow windows designed for the targeted peptides capture the isotopic peaks. This provides enhanced signal and quantification accuracy. The absolute concentrations of the target peptides are determined by calculating their light-to-heavy ratios of fragment-ion chromatograms and interpolating these values against external calibration curves derived from known standards. (C) The DIA nature of IsoPS-DIA, which covers a range of *m*/*z* sequentially allowing us to obtain the protein identification and relative abundance comparisons. Protein quantities are inferred from the peak areas of MS2 fragment-ion chromatograms.

For demonstration, our model study focused on clinically relevant *EGFR* mutations responsive to targeted therapies: point mutation L858R (exon 21, 39–47%), E746–A750 (accounting for ∼69% of exon 19 deletions, abbreviated Del19), and another common mutation G719X (X = A, C, S, D, exon 18, 2–3%).^35–38^ Six NSCLC cell lines representing these genotypes (H3255-L858R, CL97-G719A/T790M, H1975-L858R/T790M, PC9-Del19, CL68-Del19/T790M, and A549-*EGFR* WT/KRAS-G12S) were selected, collectively covering 81–99% of clinical *EGFR* mutations. In-silico digestion identified LysC and GluC as optimal proteases to generate mutant- and wild-type-containing peptides with favorable MS detectability^39^ (Table S2). For absolute quantification, heavy-isotope-labeled peptides (^13^C/^15^N-labeled C-terminal lysine or glutamic acid) were spiked as internal standards to optimize DIA settings, evaluate quantitation performance, and construct spectral libraries.

To streamline window configuration, we developed an interactive web-based IsoPS-DIA design tool on GitHub (https://github.com/Isaac-Chiu/IsoPS-DIA-Window-designer). The Python tool allows users to customize acquisition windows based on precursor *m*/*z* and retention time with an R script provided to prepare input files from Skyline exports. Using this tool, we designed 36 windows across 400–1000 *m*/*z*, including 16 narrow targeted windows (4 Th) for eight isotopic pairs of *EGFR* wild-type, L858R, G719A, and Del19 peptides, and 20 broader windows for untargeted global profiling. For instance, L858R light (454.25 *m*/*z*) and heavy (458.26 *m*/*z*) peptides were placed in adjacent 453–457 and 457–461 *m*/*z* windows, enabling independent fragmentation to collect full sets of fragment ions for quantification (Figure 1B). Full window details are provided in Supporting Table S1.

### Enhanced Detection and Quantitation Performance by IsoPS-DIA

We first evaluated the performance of fragment-ion detection of mutant peptides using IsoPS-DIA versus conventional DIA with a fixed scanning window (Fix-DIA, 15 Th). Light and heavy synthetic peptides (25 fmol) spiked into 100 ng of mouse tissue digests (with iRT peptides) were analyzed. The feature to detect full series of fragments in IsoPS-DIA was demonstrated in the example of *EGFR* Del19 peptides (^746^ELREATSPK^754^) (Figure 2A). Fix-DIA coisolated the light (515.78 *m*/*z*) and heavy (519.79 *m*/*z*) precursors within the 505–520 *m*/*z* window, producing shared *y*- and *b*-ions and leaving only a few unique low-abundance *y*-ions (y3- y6, y8) suitable for quantification (Figure 2B); only y3 and y8 showed >5% relative intensity, which limited their utility for robust quantification. In contrast, IsoPS-DIA separated the light and heavy peptides into adjacent windows, enabling independent fragmentation to detect a full series of fragments. This resulted in 10 unambiguous and quantifiable fragment ions (b3–b7, y3–y6, and y8) (Figure 2C,D). Compared to Fix-DIA, IsoPS-DIA yielded a 5-fold increase in quantifiable fragment ions, with all y-ions exhibiting 3 to 47% higher intensities.

**Figure 2:**
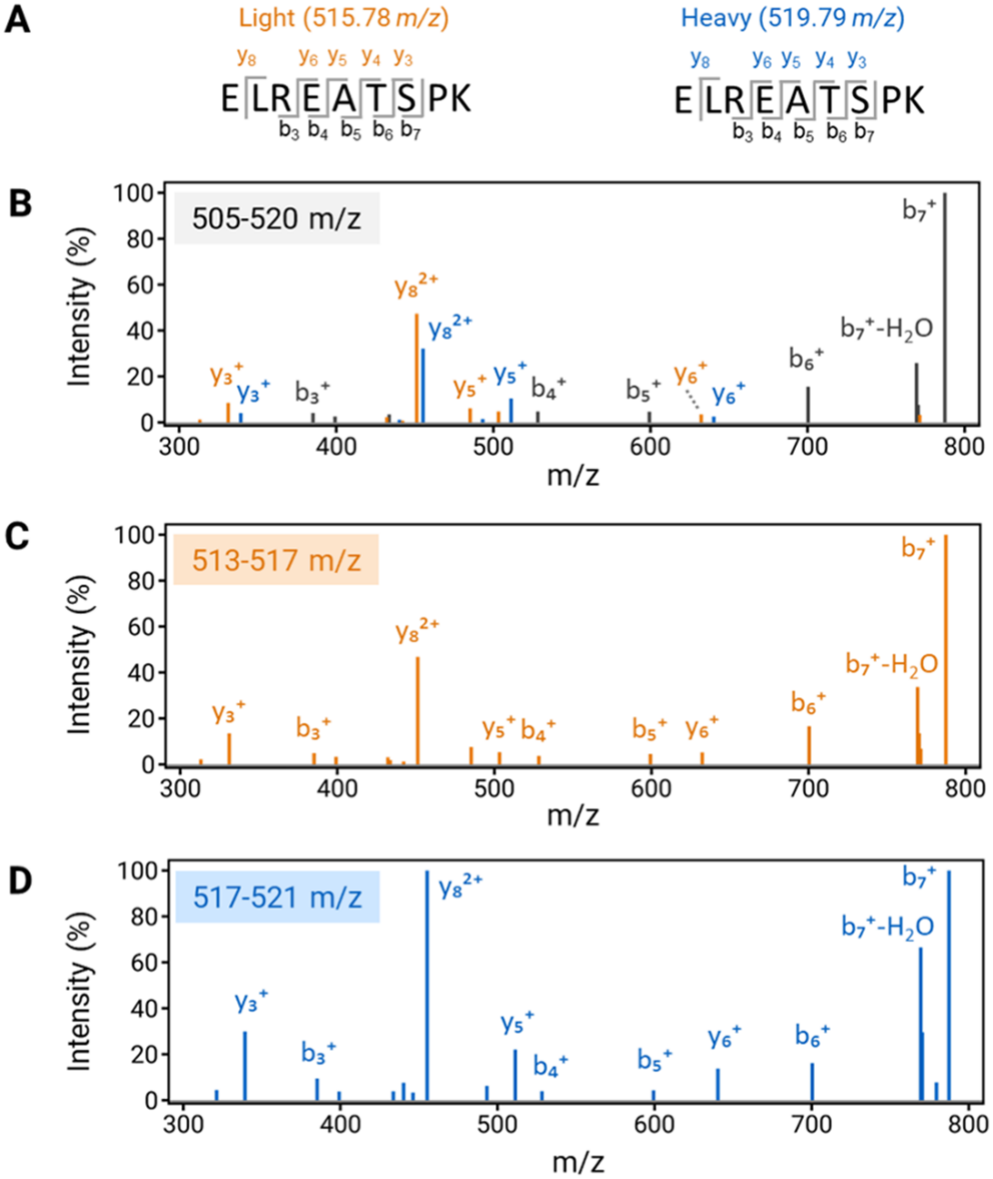
Comparison of representative light and heavy DIA-MS2 spectra of EGFR-E746-A750 wild-type peptide in conventional DIA and IsoPS-DIA. (A) Sequence of the EGFR-E746-A750 wild-type peptide and its heavy peptide with isotopic labeling on C-terminal lysine. (B) The DIA-MS2 spectrum of the wild-type peptide was acquired in the scanning window of 505–520 *m*/*z* by conventional DIA (15 Th window). The separate DIA-MS2 spectra of (C) light peptide (513–517 *m*/*z*) and (D) heavy peptide (517–521 *m*/*z*) were obtained by the IsoPS-DIA method. Both spectra show all fragments in light and heavy peptides. The unique y-ions between the isotope pair are labeled with red and blue, while the common b-ions are labeled with black.

We next benchmarked IsoPS-DIA against PRM, Fix-DIA, and Var-DIA across three concentration levels (0.75, 2.5, and 7.5 fmol) of synthetic peptides spiked into mouse lung digests. As expected, PRM showed the highest signal-to-noise ratios (SNR) for extracted ion chromatograms (XICs), and IsoPS-DIA achieved comparable profiles (Figure 3A). Among the DIA methods, IsoPS-DIA exhibited superior fragment peak profiles with reduced background interference. For the EGFR-G719A mutant and wild-type peptides, SNRs reached 21.4 and 12.5 with IsoPS-DIA, compared to 1.7 and 2.7 with Fix-DIA, and 6.3 and 6.7 with Var-DIA, respectively (Figure 3A). Across all targeted peptides, IsoPS-DIA improved median SNRs by 1.4–2.9-fold over Fix-DIA and 1.9–2.5-fold over Var-DIA (Figure 3B). Notably, at the lowest concentration (0.75 fmol), all seven target peptides were detected with higher SNRs by IsoPS-DIA, whereas 6–7 peptides fell below LOQ (SNR < 10) with Fix-DIA or Var-DIA (Figure 3C and Table S3). For four peptides, IsoPS-DIA SNRs exceeded other methods by >2-fold.

**Figure 3:**
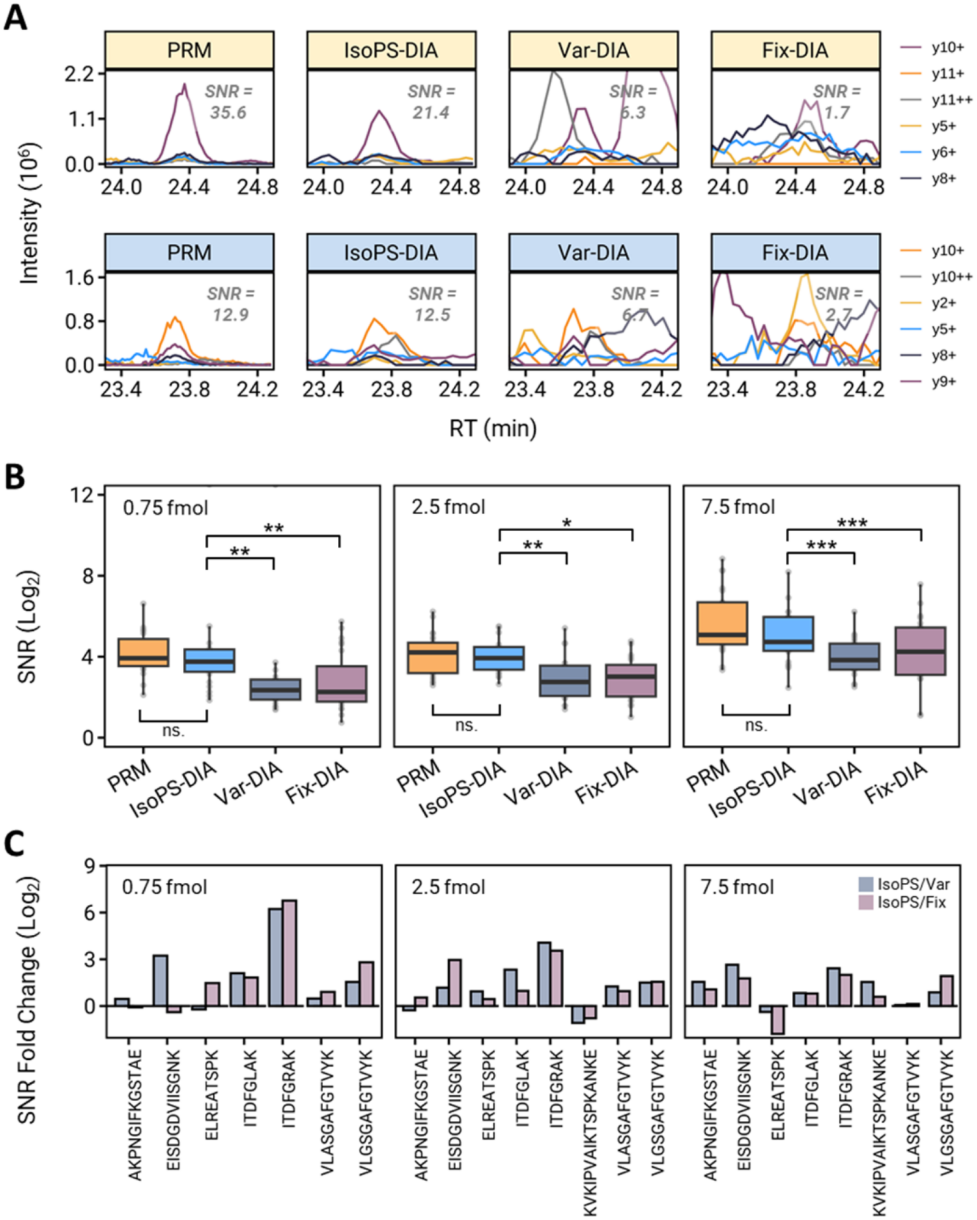
Assessment of signal-to-noise ratios (S/N) of synthetic EGFR peptides. (A) Extracted ion chromatograms (XIC) of G719 wild-type (upper panel) and G719A mutant peptides (lower panel) by PRM and 3 types of DIA settings. (B) The median S/N of all 8 EGFR light peptides using three different peptide amounts (1, 5, 25 fmol) increased when using an IsoPS window design compared with Var-DIA, and Fix-DIA. (C) Bar plots showing the S/N fold change of each peptide for both Var-DIA and Fix-DIA relative to that of IsoPS-DIA across three peptide amounts.

Absolute quantification performance was then assessed by using calibration curves constructed from light peptides (0–25 fmol) spiked with heavy internal standards (25 fmol). Two quality control (QC) samples, 2.5 and 7.5 fmol, were prepared to evaluate the absolute quantification performance. All methods achieved good linearity (average *R* ^2^ > 0.99), though Fix-DIA showed slightly lower linearity (Table S4). Notable deviation was observed for peptide ^455^EISDGDVIISGNK^467^ due to background interference in low calibrators, observed in the 0, 1, and 5 fmol calibrators (Figure S1). At the 7.5 fmol QC level, conventional Fix-DIA showed a pronounced deviation for the ^455^EISDGDVIISGNK^467^ peptide (127% RE; Figure S2A), whereas the other approaches had lower error ranges (Table S5). At the 2.5 fmol QC level, the four methods exhibited comparable accuracy, with average relative errors (REs) of 10.6, 17.8, 21.3, and 15% for Fix-DIA, IsoPS-DIA, Var-DIA, and PRM, respectively. Among the analytical merits, varied degrees of reproducibility were observed among the four methods. IsoPS-DIA demonstrated markedly superior reproducibility, particularly at low concentrations, as reflected by the low CVs at the 2.5 fmol QC level (2.2%), which is comparable to PRM (2.9%) and outperformed the other DIA approaches (Var-DIA: 15.3%, Fix-DIA: 30.3%) (Figure 4C).

**Figure 4:**
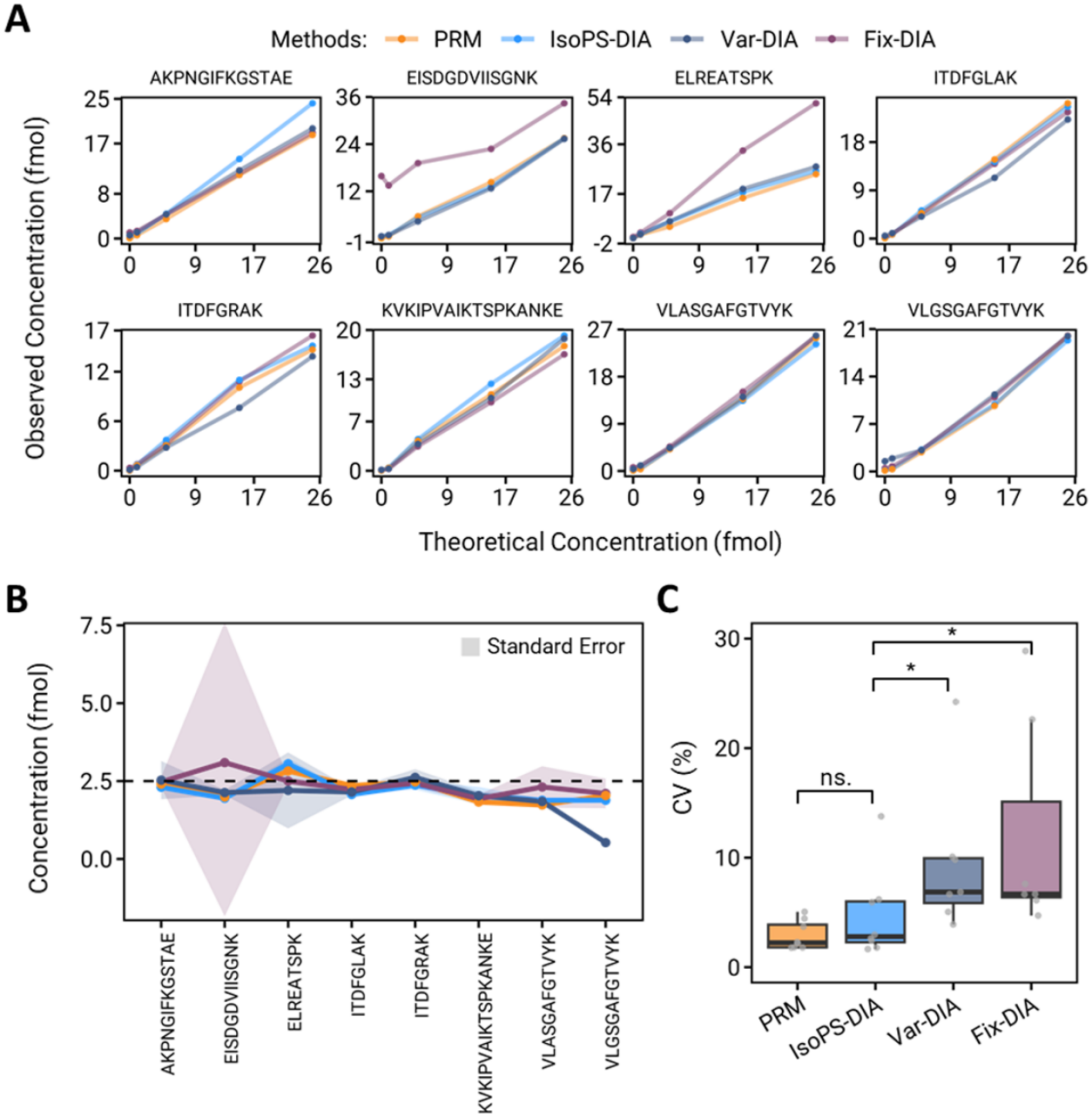
Evaluation of the quantitative performance of PRM, IsoPS-DIA, and Var- and Fix-DIA. (A) Calibration curves of the eight targeted peptides constructed by the four MS methods. (B) The absolute quantification results of 2.5 fmol spiked sample analyzed by PRM (Orange), IsoPS-DIA (Sky-blue), Var-DIA (Dark-blue), and Fix-DIA (Purple). The dashed line indicates the theoretical value (2.5 fmol). (C) The CV value obtained from the three replicate quantification results of the four methods.

In summary, IsoPS-DIA achieved PRM-like sensitivity while maintaining DIA’s broad proteomic coverage. Its narrow isolation windows enhance fragment-ion yield, SNR, and reproducibility, especially at low targeted protein concentrations.

### IsoPS-DIA Quantified Endogenous *EGFR* Mutant and Downstream Signaling Proteome

After performance validation, IsoPS-DIA was applied to six NSCLC cell lines: five harboring *EGFR* mutations (H3255–L858R, CL97–G719A/T790M, H1975–L858R/T790M, PC9–Del19, CL68–Del19/T790M) and A549 cells with wild-type *EGFR* and *KRAS*-G12S. A spectral library was built from synthetic *EGFR* mutant peptides (L858R, G719A, Del19), which were not previously reported in public repositories. Cosine similarity scores were used to distinguish wild-type and mutant peptides, which showed <20% spectral similarity in DIA data (Figure S3). All targeted peptides were confidently identified in their respective cell lines by using the library. For example, the L858R peptide (^853^ITDFGRAK^860^) was confidently detected in H3255 with high spectral similarity to its synthetic standard (Ldotp = 0.902, Figure S4A, Table S6), and Del19 peptide (^737^KVKIPVAIKTSPKANKE^753^) in CL68 cells (Ldotp = 0.823 and consistent coeluting profiles, Figure S4B). Wild-type EGFR peptides (L858, E746-A750) and other variants (G719A, G719-WT) also showed strong spectral matches (Ldotp >0.81) and consistent coelution profiles (Figure S4C–F). Importantly, the use of narrow scanning windows in IsoPS-DIA provided richer fragment patterns. Low spectral similarity (<20%) between wild-type and mutant peptides supported high specificity in their identification and quantification.

Absolute quantification was then performed to determine mutant-to-wild-type EGFR and KRAS ratios by calibration curves across 0.5–500 fmol (*R* ^2^ = 0.998–0.999) (Figure S5). IsoPS-DIA achieved subfemtomole sensitivity with LODs ranging from 36 to 222 amol and LOQs from 121 to 741 amol (Table S7) and high reproducibility with median CV = 2.4% (Table S8). Quantification revealed distinct expression patterns across the cell lines (Figure 5A). In H3255, L858R was quantified at 84.97 ± 2.84 fmol/μg versus 27.16 ± 0.76 fmol/μg WT (mutant-to-WT ratio = 3.1), while H1975 showed much lower L858R concentration (1.22 ± 0.04 vs 4.82 ± 0.10, ratio = 0.3), reflecting >10-fold variation. For the Del19 deletion, CL68 showed higher mutant expression (28.70 ± 1.01 vs 12.41 ± 0.27 fmol/μg), whereas PC9 expressed mutant and WT at comparable levels (9.49 ± 0.25 vs 8.55 ± 2.03 fmol/μg). G719A was dominantly expressed in CL97 (34.09 ± 0.75 vs 15.74 ± 0.13 fmol/μg WT). IsoPS-DIA’s multiplexing capability also enabled quantification of *KRAS* and its G12S variant, a hotspot in ∼4.4% of lung cancers.^40,41^ In A549 cells, both WT and G12S forms were detected at comparable levels (7.58 ± 0.08 vs 6.67 ± 0.06 fmol/μg) (Figure 5B). To the best of our knowledge, this is the first report of simultaneous absolute quantification of multiple endogenous *EGFR* driver mutations with their wild-type counterparts.

**Figure 5:**
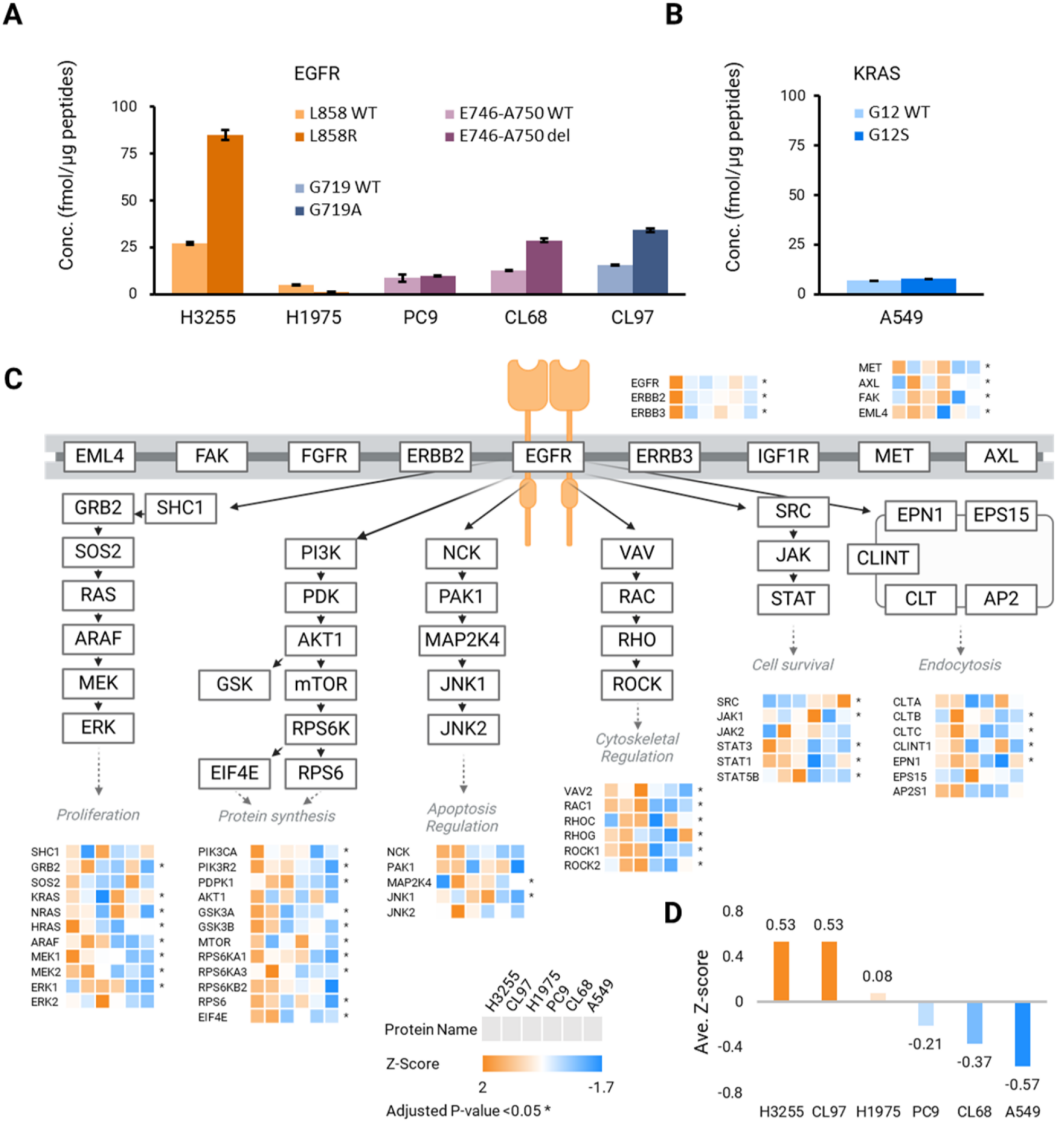
Absolute quantification of the targeted mutant peptides in 6 NSCLC cell lines and the EGFR-related comparative pathway analysis among the 6 NSCLC cell lines. (A) Absolute quantification of the mutant peptides in KRAS mutation cell line A549 (G12S) and EGFR mutation cell lines: H3255 (L858R), H1975 (L858R/T790M), PC9 (E746-A750del), CL68 (E746-A750del/T790M), and CL97 (G719A/T790M) cells. (B) Comparison of EGFR-related pathway; the Ras-Raf, PIK3-AKT, JNK, Rho-Rock, JAK-STAT, and endocytosis pathway among 6 NSCLC cell lines. (C) Comparison of the average z-score of the 6 major EGFR-related pathways in 6 NSCLC cell lines.

To evaluate how allele-specific protein expression corresponds to genomic mutation burden, we quantified the ratios of the absolute concentration of mutant proteins across six cell lines and compared them with their respective genomic variant allele frequencies (VAFs) obtained by MassARRAY (Table S8). Overall, all mutant proteins were confidently quantified by IsoPS-DIA, demonstrating the method’s ability to directly measure functional, expressed oncoproteins. Genomic VAFs were uniformly high (>70–95%) across EGFR mutant lines (H3255, H1975, PC9, CL68, CL97) and KRAS mutant A549, reflecting clonal or near-clonal mutations. In contrast, protein-level mutant ratios were systematically lower than genomic VAFs showed substantial variability (20–76% for EGFR mutants; 53% for KRAS-G12S), indicating that the protein expression of mutant vs wild-type alleles is not strictly proportional to DNA VAF. The 46% discrepancy observed in KRAS-G12S (A549) likely results from sequence homology between Ras isoforms (HRAS and NRAS) at residues 6–16, which inflates the wild-type peptide signal. Notably, high EGFR expressors (H3255, CL68, and CL97) have similar VAF between gene and protein levels (<20% discrepancies), whereas low-expressors (H1975 and PC9) exhibited discrepancies between 43 and 54%. Powered by multiprotease digestion and IsoPS-DIA, this study provides the first absolute quantification of mutant protein ratios in these isogenic lines. These findings suggest differential transcription, translation, or protein stability between alleles, highlighting that genomic mutation burden does not fully predict the abundance of the mutant protein actually present in cells. Protein-level VAF reveals functional mutant oncoprotein expression not predicted by DNA VAF, improving assessment of therapeutic target abundance.

Beyond target quantification, IsoPS-DIA enabled global proteome profiling to map the downstream signaling proteome. Using direct DIA analysis with Spectronaut, 6,183 proteins were quantified across all cell lines, including 48 components of EGFR-related signaling pathways (Ras-Raf-MEK-ERK, PI3K-AKT-mTOR, JNK, Rho-ROCK, SRC-JAK-STAT) (Figure 5C). Distinct expression patterns reflected pathway activation status: L858R mutant lines showed stronger downstream signaling. For example, H3255 exhibited high ErbB family protein levels (EGFR, ERBB2, and ERBB3), consistent with elevated L858R expression and aggressive proliferative signaling. These findings agree with the reports that L858R tumors are more aggressive than Del19 deletions.^42^ Pathway Z-scores confirmed higher relative enrichment scores for EGFR-related pathways in H3255 and CL97 cells compared to those in H1975 cells (Figure 5D), correlating with EGFR mutant abundance. In contrast, A549 displayed lower Z-scores and reduced expression of proteins in the EGFR pathway. These findings support a direct association between EGFR mutation abundance and protein expression of enriched downstream pathways.

In summary, the abundance and type of *EGFR* or *KRAS* mutation strongly influence the downstream protein expression levels of NSCLC EGFR-related signaling pathways. IsoPS-DIA uniquely provides absolute quantification of drug targets together with global pathway profiling, enabling functional stratification of oncogenic signaling and revealing actionable vulnerabilities.

### Quantitative Global Profiling of EGFR Genotype-Dependent Proteomic Landscape in NSCLC Cell Lines

Finally, we benchmarked the global profiling performance of IsoPS-DIA, Fix-DIA, and Var-DIA using six PC9 cell lines. All methods were configured with 40 MS2 events covering the 400–1000 *m*/*z* range. Despite IsoPS-DIA allocating more windows to separate isotopic paired light and heavy peptides, it provided comparable identifications using direct DIA search: 3936 protein groups for Fix-DIA, 4,130 for Var-DIA, and 4,091 for IsoPS-DIA and precursor ions (Fix-DIA: 40,577; Var-DIA: 40,215; IsoPS-DIA: 34,673) in 100 ng mouse tissue peptide samples (Figure S6A,B). Importantly, triplicate analyses of IsoPS-DIA achieved superior quantification reproducibility, with 78% of protein groups showing CVs < 10%, compared to 60% for Fix-DIA and 63.7% for Var-DIA (Figure S6A). Triplicates also showed high correlations (Spearman *ρ* > 0.995; Figure S9C), indicating that narrower windows in IsoPS-DIA do not compromise proteome coverage or sensitivity.

We next applied IsoPS-DIA to six NSCLC cell lines, identifying 6183 protein groups in total, with 5357–5735 proteins detected per single-shot run using direct DIA search (Figure 6A). Differentially expressed proteins (DEPs) were determined after normalization, imputation, and statistical filtering (Kruskal–Wallis H test, adjusted *p* < 0.05), followed by hierarchical clustering. As shown in Figure 6B, PC9 and CL68, both harboring Del19 deletions, clustered together and exhibited similar proteomic profiles compared to A549 (KRAS-G12S mutation), while H3255 (L858R) and CL97 (G719A) form another cluster. H1975, with the lowest EGFR expression and mutant-to-wild-type protein expression ratio, displayed a distinct proteomic signature (Figure 6B).

**Figure 6:**
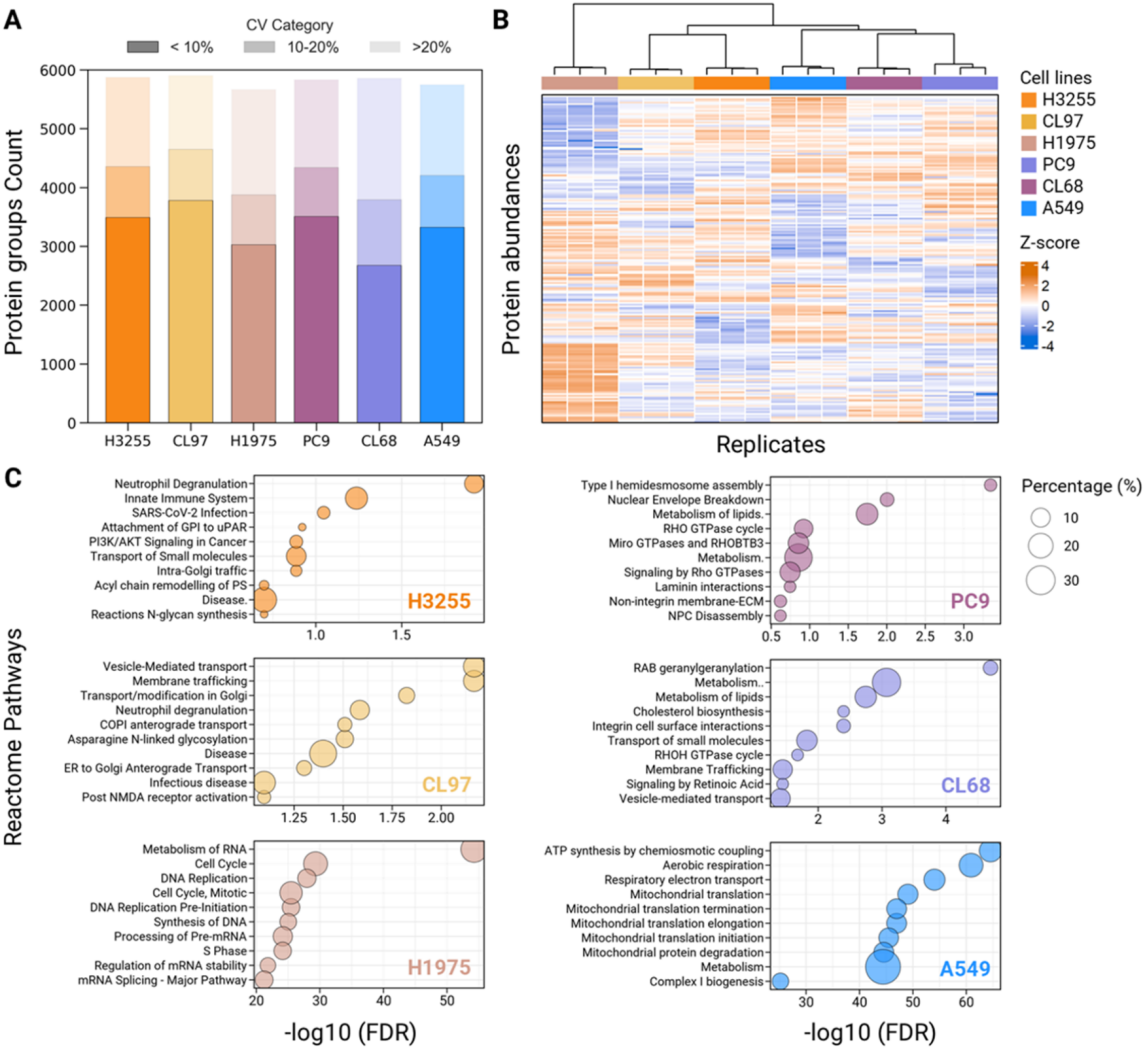
Summary of protein identification and quantitative comparison of 6 NSCLC cell lines. (A) Comparison of number of identified proteins in H3255 (EGFR-L858R), CL97 (EGFR-G719A/T790M), H1975 (EGFR-L858R/T790M), PC9 (EGFR-E746-A750del), CL68 (EGFR-E746-A750del/T790M), and A549 (KRAS-G12S) cells with the CV categories. (B) Unsupervised clustering results of differential expression pattern. (C) Top 10 enriched pathways (adjusted p-value <0.05) by KEGG pathway enrichment analysis for the differentially expressed proteins among 6 cell lines.

These clustering patterns were concordant with pathway-level comparisons (Figure 5D), demonstrating the agreement between global proteomic shifts and enriched EGFR-related signaling pathways. In total, 3166 DEPs were identified across the six cell lines (Wilcoxon signed-rank, adjusted *p* < 0.05). Reactome pathway enrichment analysis of upregulated proteins revealed the top ten enriched pathways for each cell line (Figure 6C). H3255 cells exhibited enrichment in PI3K-AKT-related pathways, while CL97 cells showed enrichment in endocytosis-related processes. The analysis further highlighted common functional categories, such as membrane trafficking, vesicle-mediated transport, and ER/Golgi-associated pathways, in these cell lines, reinforcing the complementary insights gained from IsoPS-DIA. In addition, distinct signatures were also observed: H1975 displayed strong enrichment in cell cycle and RNA metabolism pathways (FDR – log10 > 60), while A549 showed mitochondrial pathway activation—reflecting EGFR-independent signaling alterations in NSCLC.

To demonstrate the general applicability of IsoPS-DIA across different instrument platforms, we applied the method for the three of the same cell lines using a Q-TOF instrument (TripleTOF 5600). The calibration curves of seven EGFR peptides showed excellent linearity with *R* ^2^ values exceeding 0.993 (Figure S7A). We further quantified the L858R mutation peptide and its corresponding wild-type peptide in A549, H3255, and PC9 cell lines, obtaining 80 fmol per μg peptide from cell sample from L858R-bearing H3255 cell. The overall expression levels are consistent with those measured on the Orbitrap platform (Figure S7B). In addition, DIA analysis enabled the identification of 1872, 1316, and 2330 proteins in A549, H3255, and PC9 cells, respectively (Figure S7C). Together, these results highlight the compatibility of the IsoPS-DIA strategy with the Q-TOF instrument.

In summary, IsoPS-DIA uniquely integrates the absolute quantification of EGFR and KRAS mutant proteins with unbiased global proteomic profiling. While genotypes are defined by DNA sequencing, our results revealed marked variability in mutant versus wild-type protein abundance, suggesting allele-specific expression that may affect the therapeutic response. In parallel, downstream proteomic profiling uncovered functional pathway alterations, providing a systems-level framework to evaluate tumor draggability beyond the genotype alone.

## Conclusions

In this study, IsoPS-DIA was developed to offer dual functionality in a single LC-MS/MS run: (i) multiplexed absolute quantification with strength to distinguish mutations and (ii) simultaneous global proteome profiling to read out downstream signaling and resistance pathways. Methodologically, the key advance is the dual-window scheme: narrow 4 Th windows resolve light/heavy pairs into adjacent scans, eliminating coisolation and overlapping fragment spectra that confound isotope-dilution DIA, while wide variable windows preserve proteome coverage. This design yields richer unique fragments and higher S/N, enabling subfmol sensitivity of LODs (36–222 amol), low LOQs (121–741 amol), high linearity (*R* ^2^ ≈ 0.998–0.999), and low CVs (∼2–3%), approaching PRM-like precision without sacrificing DIA-level breadth of >5000 proteins (dependent on the instrument).

Compared with SRM/PRM, which remains the gold standard for absolute quantification but scales poorly and lacks global proteome information, IsoPS-DIA retains high multiplexing and system-wide readouts. Relative to Hybrid-DIA and mixed DIA–PRM modes that alternate or trigger PRM scans, our approach does not rely on instrument-specific APIs or duty-cycle trade-offs for targeted events, and it directly supports absolute (not just relative) quantification. IsoPS-DIA’s isotope separation removes spectral deconvolution ambiguity for light and heavy peptides and increases the number and intensity of quantifiable ions. Thus, IsoPS demonstrated improved accuracy at low abundance—critical for endogenous variant peptides and combined with a multiprotease strategy to maximize mutant site detectability for distinguishing mutant vs wild-type forms. Practically, IsoPS-DIA is applicable to different platforms (Orbitrap/Q-TOF) and uses standard software. To facilitate adoption, we developed an open-source, web-based IsoPS-DIA window-design tool (https://github.com/Isaac-Chiu/IsoPS-DIA-Window-designer), written in Python and freely available to the community. The tool customizes DIA scanning windows based on precursor *m*/*z* and retention time with an *R* script for preparing inputs from Skyline exports. By releasing this resource, we aim to facilitate broad adoption of IsoPS-DIA and its application to mutant proteins, as well as other targeted or discovery proteomics studies.

Application of IsoPS to NSCLC cell lines enabled the first direct quantification of endogenous EGFR mutant and wild-type proteins, revealing heterogeneous expression patterns and mutation-to-wild-type ratios that cannot be inferred from gene testing data alone. These findings highlight the value of protein-level measurements to uncover allele-specific expression and post-transcriptional regulation, which are critical for predicting therapeutic responses to EGFR-targeted therapies. This IsoPS strategy established a reproducible and scalable assay as a powerful complement to conventional gene testing in clinical and translational research.

While IsoPS-DIA performed robustly for 16 peptides tested, limitations include a finite number of narrow windows per run (target-set sizing and duty-cycle schedule). Advancement in high-speed mass spectrometers will help scale up to larger target panels or make strategic adjustments to maintain analytical quality. The latter may include optimizing window schemes through multiplexing, adjusting *m*/*z* coverage, or increasing the LC gradient time. Given its narrow-window design, IsoPS-DIA is optimally suited for panels of 25–35 target peptides in light–heavy pairs. Future work will focus on extending this platform to broader target sets and clinical sample types, enabling comprehensive quantitative readouts of oncogenic mutations and their functional proteomic consequences.

## Supplementary Material





## Data Availability

Mass spectrometry raw files are available on jPOST database^43^ (https://globe.jpostdb.org/) with the jPOST ID: JPST003737 and the PXID: PXD068094.

## Notes

LC-MS/MS Method for IsoPS-DIA in Q-TOF Mass Spectrometer; the XICs of ^455^EISDGDVIISGNK^467^ peptide from Fix-DIA, IsoPS-DIA, PRM, and Var-DIA (Figure S1); quantitation performance of 7.5 fmol spiked peptides (Figure S2); comparison of spectral similarity between DDA and DIA identification result for 3 pairs of EGFR wild-type and mutant peptides (Figure S3); identification of endogenous targeted peptides in NSCLC cell lines (Figure S4); quantification performance of EGFR and KRAS peptides across dilution series (Figure S5); comparison of proteome profiling coverage of PC9 by Fix-DIA, IsoPS-DIA, and Var-DIA methods (Figure S6); summary of targeted quantitation and proteome profiling results in A549, H3255, and PC9 by IsoPS-DIA with Q-TOF systems (Figure 7) (PDF)
Summary of DIA window size and range (Table S1); list of target peptide sequence of EGFR (Table S2); comparison of signal-to-noise ratio of peptides detected by 4 methods (Table S3); summary of calibration curves for each peptides obtained by 4 methods (Table S4); (Table S4), and summary of quantitation results of each peptide by 4 methods (Table S5); summary of spectra similarity between identified peptides and lpeptide sin the spectra ibrary (Table S6); summary of LOD and LOQ (Table S7); summary of quantified ratio of mutant protein and their corresponding gene variant allel frequency (VAF) across 6 cell lines (Table S8) (XLSX).

## #

University of Wisconsin–Madison: Madison, Wisconsin, US

## References

[1] Mitsudomi T., Suda K., Yatabe Y. (2013). Surgery for NSCLC in the era of personalized
medicine. Nat. Rev. Clin Oncol.

[2] Skoulidis F., Li B. T., Dy G. K., Price T. J., Falchook G. S., Wolf J., Italiano A., Schuler M., Borghaei H., Barlesi F. (2021). Sotorasib
for Lung Cancers with KRAS p.G12C Mutation. N. Engl. J. Med..

[3] Bronte G., Rizzo S., Paglia L. L., Adamo V., Siragusa S., Ficorella C., Santini D., Bazan V., Colucci G., Gebbia N., Russo A. (2010). Driver mutations and differential
sensitivity to targeted therapies: a new approach to the treatment
of lung adenocarcinoma. Cancer Treat. Rev..

[4] Yoshikawa S., Kukimoto-Niino M., Parker L., Handa N., Terada T., Fujimoto T., Terazawa Y., Wakiyama M., Sato M., Sano S. (2013). Structural basis for the altered drug sensitivities
of non-small cell lung cancer-associated mutants of human epidermal
growth factor receptor. Oncogene.

[5] Harvey W. T., Carabelli A. M., Jackson B., Gupta R. K., Thomson E. C., Harrison E. M., Ludden C., Reeve R., Rambaut A., Peacock S. J., Robertson D. L. (2021). SARS-CoV-2 variants, spike mutations
and immune escape. Nat. Rev. Microbiol..

[6] Arad G., Geiger T. (2023). Functional
Impact of Protein-RNA Variation in Clinical
Cancer Analyses. Mol. Cell Proteomics.

[7] Purvine S., Eppel J.-T., Yi E. C., Goodlett D. R. (2003). Shotgun collision-induced
dissociation of peptides using a time of flight mass analyzer. Proteomics.

[8] Venable J. D., Dong M.-Q., Wohlschlegel J., Dillin A., Yates J. R. (2004). Automated
approach for quantitative analysis of complex peptide mixtures from
tandem mass spectra. Nat. Methods.

[9] Gillet L. C., Navarro P., Tate S., Röst H., Selevsek N., Reiter L., Bonner R., Aebersold R. (2012). Targeted Data
Extraction of the MS/MS Spectra Generated by Data-independent Acquisition:
A New Concept for Consistent and Accurate Proteome Analysis*. Mol. Cell. Proteomics.

[10] Zhang F., Ge W., Ruan G., Cai X., Guo T. (2020). Data-Independent Acquisition
Mass Spectrometry-Based Proteomics and Software Tools: A Glimpse in
2020. Proteomics.

[11] Kitata R. B., Yang J. C., Chen Y. J. (2022). Advances in data-independent
acquisition
mass spectrometry towards comprehensive digital proteome landscape. Mass Spectrom. Rev..

[12] Lou R., Shui W. (2024). Acquisition and Analysis
of DIA-Based Proteomic Data: A Comprehensive
Survey in 2023. Mol. Cell. Proteomics.

[13] Martínez-Val A., Fort K., Koenig C., Van der Hoeven L., Franciosa G., Moehring T., Ishihama Y., Chen Y.-j., Makarov A., Xuan Y., Olsen J. V. (2023). Hybrid-DIA:
intelligent
data acquisition integrates targeted and discovery proteomics to analyze
phospho-signaling in single spheroids. Nat.
Commun..

[14] Goetze S., van Drogen A., Albinus J. B., Fort K. L., Gandhi T., Robbiani D., Laforte V., Reiter L., Levesque M. P., Xuan Y., Wollscheid B. (2024). Simultaneous targeted and discovery-driven
clinical proteotyping using hybrid-PRM/DIA. Clin. Proteomics.

[15] Sanner A., Hardt R., Matzner U., Winter D. (2024). Data-Independent Acquisition–Parallel
Reaction Monitoring Acquisition Reveals Age-Dependent Alterations
of the Lysosomal Proteome in a Mouse Model of Metachromatic Leukodystrophy. Anal. Chem..

[16] Keshishian H., Addona T., Burgess M., Mani D. R., Shi X., Kuhn E., Sabatine M. S., Gerszten R. E., Carr S. A. (2009). Quantification
of cardiovascular biomarkers in patient plasma by targeted mass spectrometry
and stable isotope dilution. Mol. Cell. Proteomics.

[17] Liu Y., Hüttenhain R., Surinova S., Gillet L. C., Mouritsen J., Brunner R., Navarro P., Aebersold R. (2013). Quantitative
measurements of N-linked glycoproteins in human plasma by SWATH-MS. Proteomics.

[18] Kim Y. J., Chambers A. G., Cecchi F., Hembrough T. (2018). Targeted data-independent
acquisition for mass spectrometric detection of RAS mutations in formalin-fixed,
paraffin-embedded tumor biopsies. J. Proteomics.

[19] Husson G., Delangle A., O’Hara J., Cianferani S., Gervais A., Van Dorsselaer A., Bracewell D., Carapito C. (2018). Dual Data-Independent Acquisition
Approach Combining
Global HCP Profiling and Absolute Quantification of Key Impurities
during Bioprocess Development. Anal. Chem..

[20] Nesvizhskii A. I. (2014). Proteogenomics:
concepts, applications and computational strategies. Nat. Methods.

[21] Rodriguez H., Zenklusen J. C., Staudt L. M., Doroshow J. H., Lowy D. R. (2021). The next
horizon in precision oncology: Proteogenomics to inform cancer diagnosis
and treatment. Cell.

[22] Manes N. P., Nita-Lazar A. (2018). Application
of targeted mass spectrometry in bottom-up
proteomics for systems biology research. J.
Proteomics.

[23] Lin T.-T., Zhang T., Kitata R. B., Liu T., Smith R. D., Qian W.-J., Shi T. (2023). Mass spectrometry-based targeted
proteomics for analysis of protein mutations. Mass Spectrom. Rev..

[24] Tan Z., Zhu J., Stemmer P. M., Sun L., Yang Z., Schultz K., Gaffrey M. J., Cesnik A. J., Yi X., Hao X. (2020). Comprehensive Detection of Single Amino Acid Variants and Evaluation
of Their Deleterious Potential in a PANC-1 Cell Line. J. Proteome Res..

[25] Chen H., Hsiao Y.-C., Chiang S.-F., Wu C.-C., Lin Y.-T., Liu H., Zhao H., Chen J.-S., Chang Y.-S., Yu J.-S. (2016). Quantitative
analysis of wild-type and V600E mutant BRAF proteins in colorectal
carcinoma using immunoenrichment and targeted mass spectrometry. Anal. Chim. Acta.

[26] Zhang Y., Bilbao A., Bruderer T., Luban J., Strambio-De-Castillia C., Lisacek F., Hopfgartner G., Varesio E. (2015). The Use of Variable
Q1 Isolation Windows Improves Selectivity in LC–SWATH–MS
Acquisition. J. Proteome Res..

[27] Han C. L., Chien C. W., Chen W. C., Chen Y. R., Wu C. P., Li H., Chen Y. J. (2008). A multiplexed quantitative strategy for membrane proteomics:
opportunities for mining therapeutic targets for autosomal dominant
polycystic kidney disease. Mol. Cell. Proteomics.

[28] Pino L. K., Searle B. C., Bollinger J. G., Nunn B., MacLean B., MacCoss M. J. (2020). The Skyline ecosystem: Informatics for quantitative
mass spectrometry proteomics. Mass Spectrom.
Rev..

[29] Stekhoven D. J., Bühlmann P. (2012). MissForest—non-parametric missing value imputation
for mixed-type data. Bioinformatics.

[30] Szklarczyk D., Kirsch R., Koutrouli M., Nastou K., Mehryary F., Hachilif R., Gable A. L., Fang T., Doncheva N. T., Pyysalo S. (2023). The STRING
database in 2023: protein-protein association
networks and functional enrichment analyses for any sequenced genome
of interest. Nucleic Acids Res..

[31] Milacic M., Beavers D., Conley P., Gong C., Gillespie M., Griss J., Haw R., Jassal B., Matthews L., May B. (2024). The Reactome Pathway Knowledgebase 2024. Nucleic Acids Res..

[32] van
der Meer D., Barthorpe S., Yang W., Lightfoot H., Hall C., Gilbert J., Francies H. E., Garnett M. J. (2019). Cell Model
Passports—a hub for clinical, genetic and functional datasets
of preclinical cancer models. Nucleic Acids
Res..

[33] Thomas R. K., Baker A. C., DeBiasi R. M., Winckler W., LaFramboise T., Lin W. M., Wang M., Feng W., Zander T., MacConaill L. E. (2007). High-throughput oncogene mutation profiling
in human cancer. Nat. Genet..

[34] Su K.-Y., Kao J.-T., Ho B.-C., Chen H.-Y., Chang G.-C., Ho C.-C., Yu S.-L. (2016). Implementation
and Quality Control
of Lung Cancer EGFR Genetic Testing by MALDI-TOF Mass Spectrometry
in Taiwan Clinical Practice. Sci. Rep..

[35] Gazdar A. F. (2009). Activating
and resistance mutations of EGFR in non-small-cell lung cancer: role
in clinical response to EGFR tyrosine kinase inhibitors. Oncogene.

[36] Hsu K.-H., Ho C.-C., Hsia T.-C., Tseng J.-S., Su K.-Y., Wu M.-F., Chiu K.-L., Yang T.-Y., Chen K.-C., Ooi H. (2015). Identification of Five Driver Gene Mutations in Patients
with Treatment-Naïve Lung Adenocarcinoma in Taiwan. PLoS One.

[37] Su J., Zhong W., Zhang X., Huang Y., Yan H., Yang J., Dong Z., Xie Z., Zhou Q., Huang X. (2017). Molecular characteristics
and clinical outcomes of
EGFR exon 19 indel subtypes to EGFR TKIs in NSCLC patients. Oncotarget.

[38] André F., The A. P. G. C., André F., Arnedos M., Baras A. S., Baselga J., Bedard P. L., Berger M. F., Bierkens M., Calvo F. (2017). AACR Project GENIE: Powering Precision Medicine through
an International Consortium. Cancer Discovery.

[39] Hoofnagle A. N., Whiteaker J. R., Carr S. A., Kuhn E., Liu T., Massoni S. A., Thomas S. N., Townsend R. R., Zimmerman L. J., Boja E. (2016). Recommendations for the Generation, Quantification,
Storage, and Handling of Peptides Used for Mass Spectrometry-Based
Assays. Clin. Chem..

[40] Aredo J., Padda S., Kunder C., Han S., Wakelee H. (2019). OA03.07 Evaluating
Racial Differences in KRAS-Mutant Non-Small Cell Lung Cancer. J. Thoracic Oncol..

[41] Gao Q., Ouyang W., Kang B., Han X., Xiong Y., Ding R., Li Y., Wang F., Huang L., Chen L. (2020). Selective targeting
of the oncogenic KRAS G12S mutant
allele by CRISPR/Cas9 induces efficient tumor regression. Theranostics.

[42] Liu J.-Y., Wang S.-Z., Yuan H.-Q., Li J.-L., Xing P.-Y. (2024). Patients
with non-small cell lung cancer with the exon 21 L858R mutation: From
distinct mechanisms to epidermal growth factor receptor tyrosine kinase
inhibitor treatments (Review). Oncol. Lett..

[43] Okuda S., Yoshizawa A. C., Kobayashi D., Takahashi Y., Watanabe Y., Moriya Y., Hatano A., Takami T., Matsumoto M., Araki N. (2025). jPOST environment accelerates
the reuse and reanalysis of public proteome mass spectrometry data. Nucleic Acids Res..

