# Supplementary material for: IsoPS-DIA: Dual Functionality of Absolute Targeted Quantification and Global Proteome Profiling
Source: Anal Chem. 2026 Jan 27;98(7):5227–38. doi: 10.1021/acs.analchem.5c05651 (PMC12937053; doi:10.1021/acs.analchem.5c05651)
Supplement: Supplementary file 1 [file ac5c05651_si_001.pdf]

Supporting Information

IsoPS-DIA: Dual Functionality of Absolute Targeted Quantification and Global Proteome Profiling

Hsin-Ju Chan[1,2,∇], Huan-Chi Chiu[1,2,∇],Li-Yu Chen[1,2], Reta Birhanu Kitata[1], Chia-Yen Wang[1,2],Shr-Uen Lin[3], Sung-Liang Yu[4,5], and Yu-Ju Chen[1,2*]

[1]Institute of Chemistry, Academia Sinica, Taipei 11529, Taiwan.

[2]Department of Chemistry, National Taiwan University, Taipei 10617, Taiwan.

[3]Graduate Institute of Oncology, College of Medicine, National Taiwan University, Taipei, Taiwan.

[4]Department of Clinical and Laboratory Sciences and Medical Biotechnology, National Taiwan University College of Medicine, Taipei, Taiwan.

[5]Department of Laboratory Medicine, National Taiwan University Hospital, Taipei, Taiwan

[∇]Hsin-Ju Chan. and Huan-Chi Chiu. contributed equally.

* E-mail: yujuchen@as.edu.tw

Table of Contents

Supporting Methods………………………………………………………………………….…3

LC-MS/MS Method for IsoPS-DIA in Q-TOF Mass Spectrometer……………………………3

Supporting Figures ……………………………………………………………………..……4

Figure S1: The XICs of ^{455}EISDGDVIISGNK^{467} peptide from Fix-DIA, IsoPS-DIA, PRM, and Var DIA……………….………………………………………………...……….……4

Figure S2: Quantitation performance of 7.5 fmol spiked peptides…………….…………..……5

Figure S3: Comparison of spectral similarity between DDA and DIA identification result for 3 pairs of EGFR wild-type and mutant peptides…………………….…………...…….….……6

Figure S4: Identification of endogenous targeted peptides in NSCLC cell lines………………7

Figure S5: Quantification performance of EGFR and KRAS peptides across dilution series...10

Figure S6: Comparison of proteome profiling coverage of PC9 by Fix-DIA, IsoPS-DIA, and Var-DIA methods………………………………………………………...……………11

Figure S7: Summary of targeted quantitation and proteome profiling results in A549, H3255, and PC9 by IsoPS-DIA with Q-TOF systems………………………………………………...12

Supporting Methods

LC-MS/MS Method for isoPS-DIA in Q-TOF mass spectrometer:

Membrane protein digests from A549, H1975, and PC9 cell lines were analyzed using the TripleTOF 5600 System (AB SCIEX Concord, ON Canada). Peptide samples reconstituted in 0.1% FA in H2O were injected into a 100 µm × 150 mm self-packed 3 µm C18-AQ column in a nanoACQUITY Ultra Performance LCTM. The mobile phases are (A) 0.1% formic acid in water and (B) 0.1% formic acid in acetonitrile (ACN). The 90-min gradient is as follows: 1–5% B in 1 min, 5–10% in 9 min, 10–25% in 48 min, 25–35% in 10 min, ramp to 85% in 1 min, wash at 85% for 5 min, and equilibrium at 1% for 14 min. Data was acquired using an ion spray voltage of 2.5 kV, curtain gas of 15 PSI, and an interface heater temperature of 150 °C. Data acquisition was done by SWATH-positive mode. MS1 settings are 390–1250 m/z, with 40 variable isolation windows (4–60 Th) the isolation window design listed in Supporting Table S1, and CID collision energy was set at 25%. MS2 scans were acquired from 100-2000 m/z.

Supporting Figures

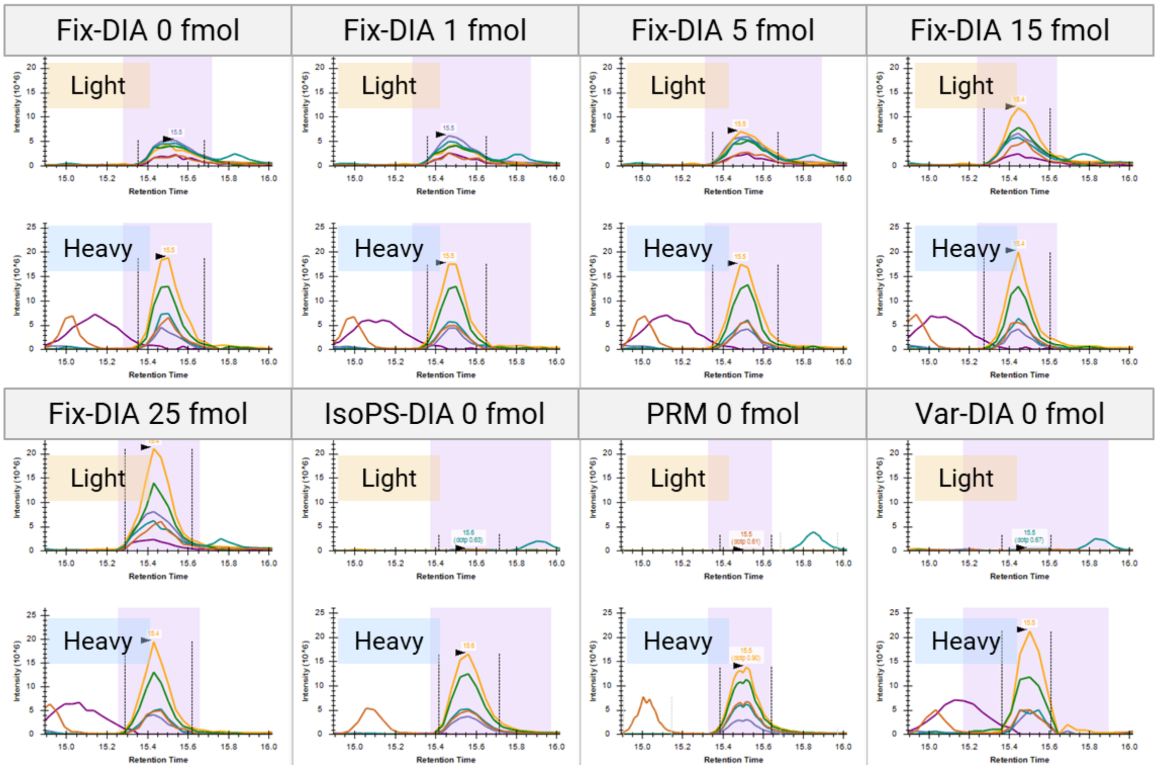

Figure S1: The XICs of ^{455}EISDGDVIISGNK^{467} peptide from Fix-DIA, IsoPS-DIA, PRM, and Var-DIA.

The XICs for 0, 1, 5, 15, and 25 fmol peptides extracted by Fix-DIAexhibited high background signals in low-concentration samples (0, 1, and 5 fmol). In contrast, IsoPS-DIA, PRM, and Var-DIA displayed no such backgrounds at 0 fmol .

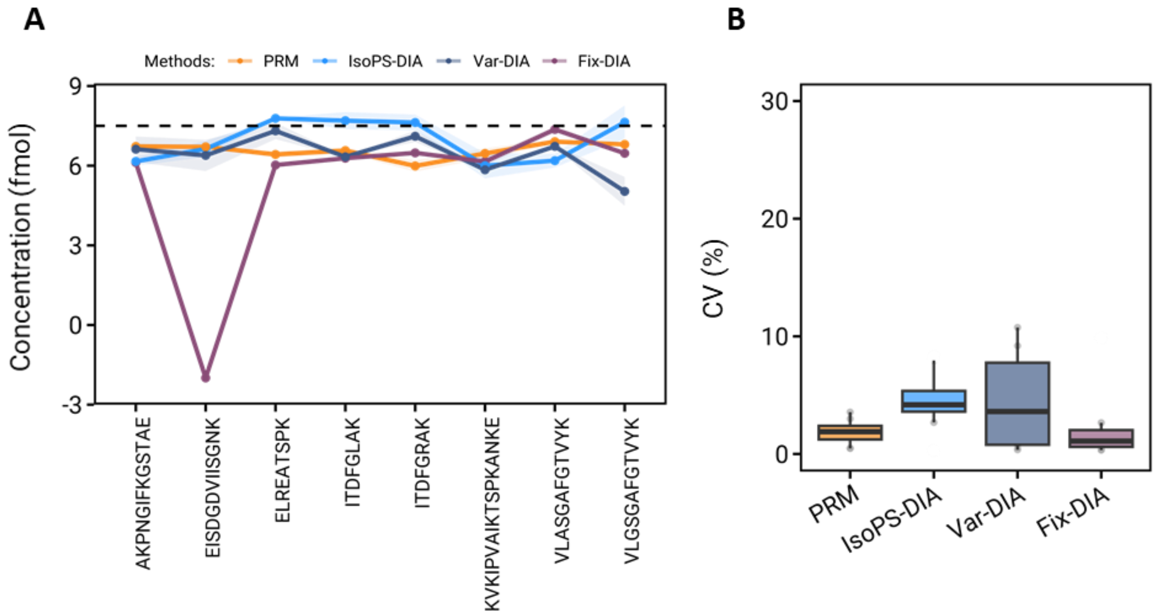

Figure S2: **Quantitation performance of 7.5 fmol spiked peptides** (A) The absolute quantification results of 7.5 fmol spiked sample analyzed by PRM (Orange), IsoPS-DIA (Sky-blue), Var-DIA (Dark-blue), and Fix-DIA (Purple). The dash line indicates the theoretical value (7.5 fmol). (B) Comparison of CV value obtained from triplicate quantification results of the four methods.

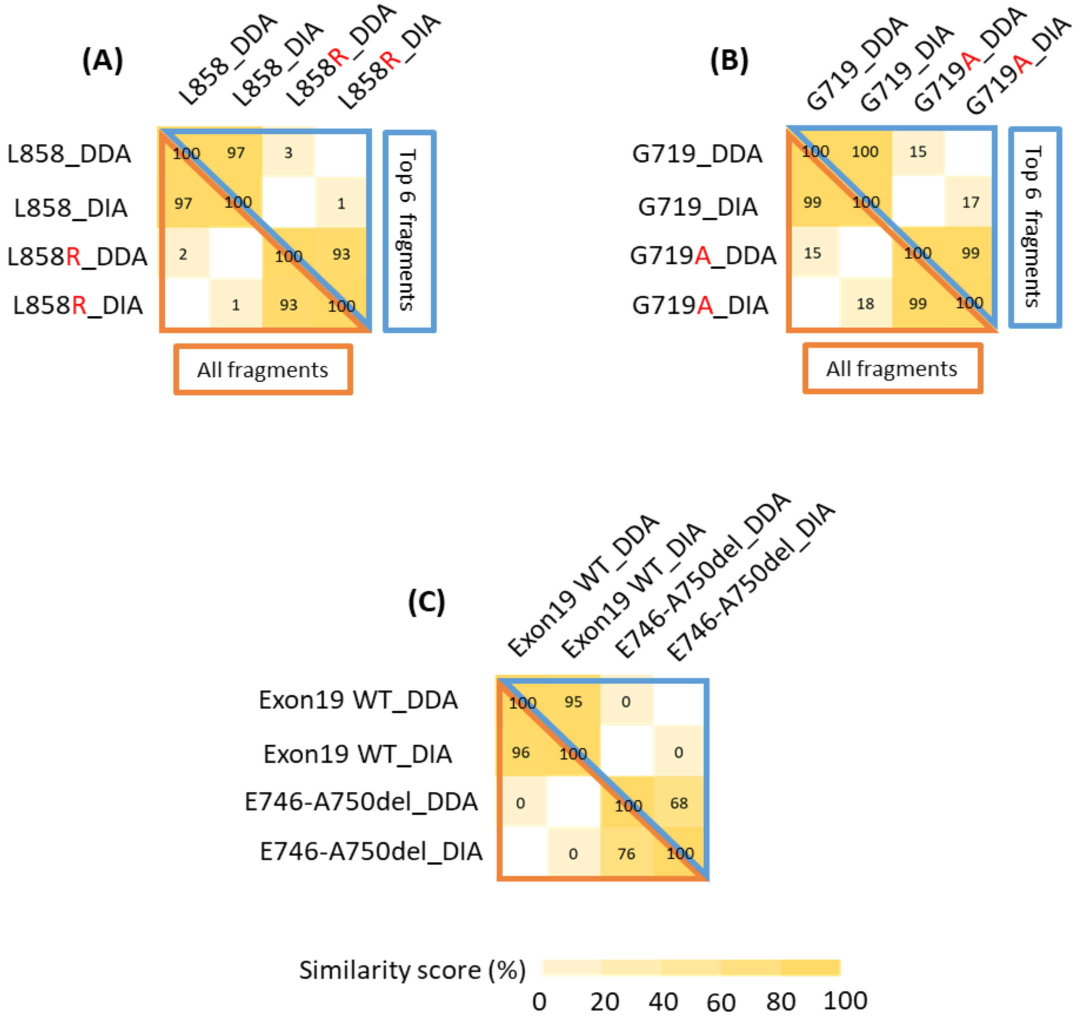

Figure S3. Comparison of spectral similarity between DDA and DIA identification results for 3 pairs of EGFR wild-type and mutant peptides.

Cosine similarity (%) between DDA and DIA identification results for (A) L858R mutation and its wild-type, (B) G719A mutation and its wild-type, and (C) E746-A750 deletion and its wild-type (C) considering all fragments (red triangle) and top 6 intense fragments (blue triangle). The similarity score was shown in percentage.

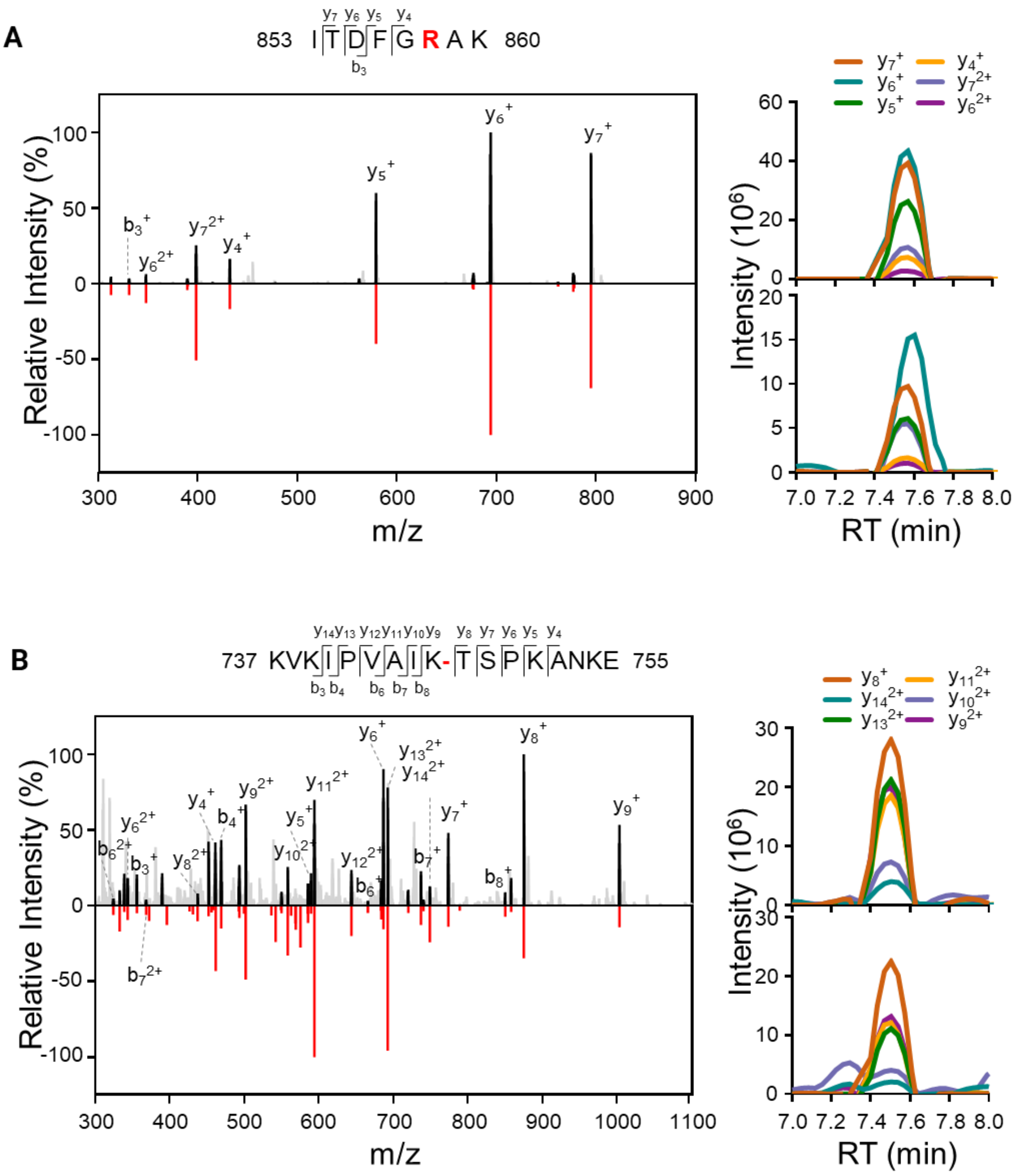

Figure S4. Identification of endogenous targeted peptides in NSCLC cell lines.

Left panels: DIA MS2 spectra of the endogenous targeted peptides are aligned to the reference spectrum in the spectral library in the left panels. Right panels: Fragment ion XICs of endogenous and spiked-in internal standard. (A) EGFR L858R peptide detected in the H3255 cell line. (B) EGFR Del19 peptide detected in the CL68 cell line.

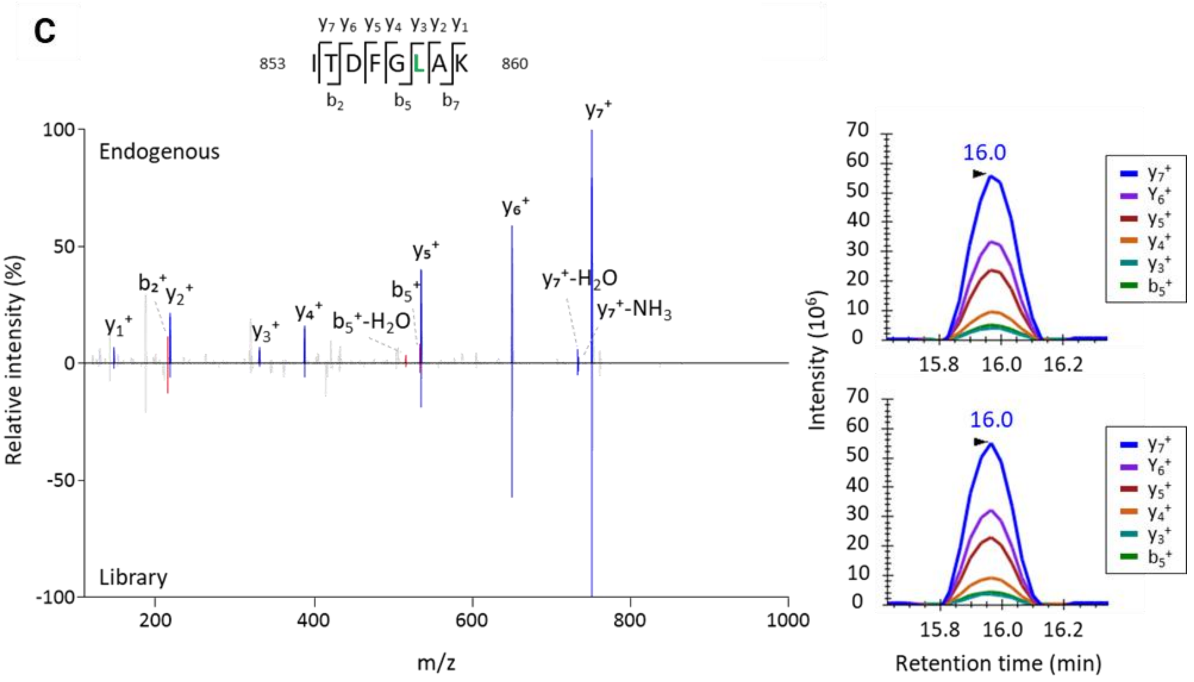

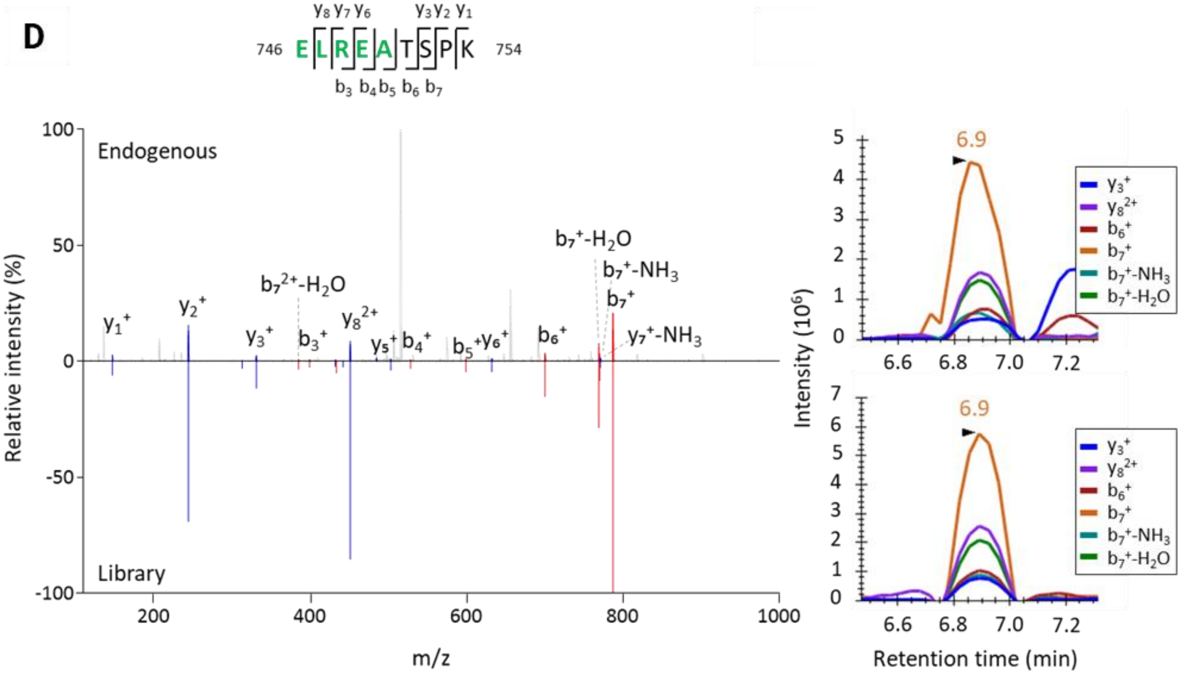

(C) EGFR L858 wild-type peptide detected in the H3255 cell line. (D) EGFR Del19 wild-type peptide detected in the CL68 cell line.

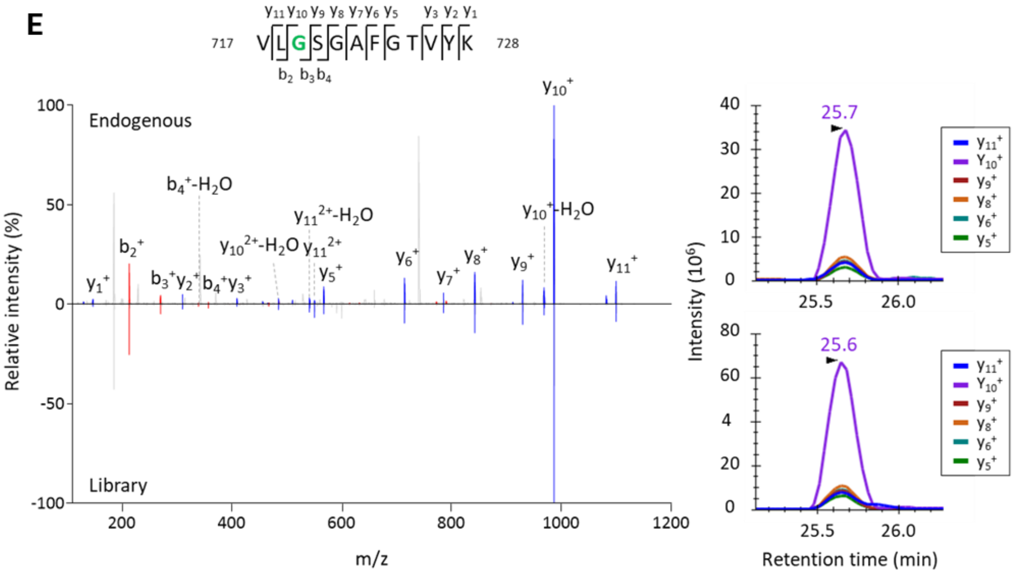

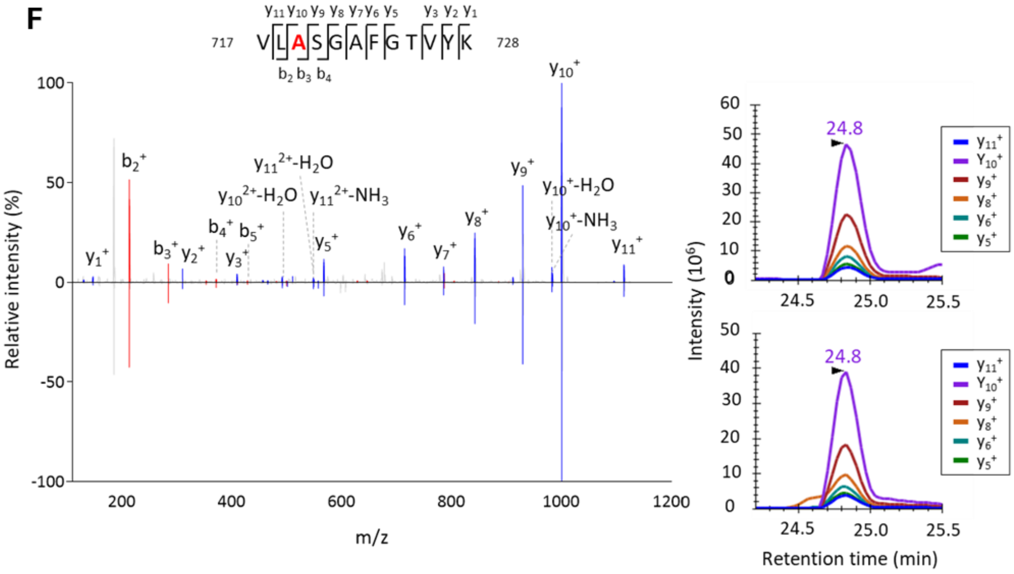

(E) EGFR G719 wild-type peptide detected in the CL97 cell line. (F) EGFR G719A peptide detected in the CL97 cell line.

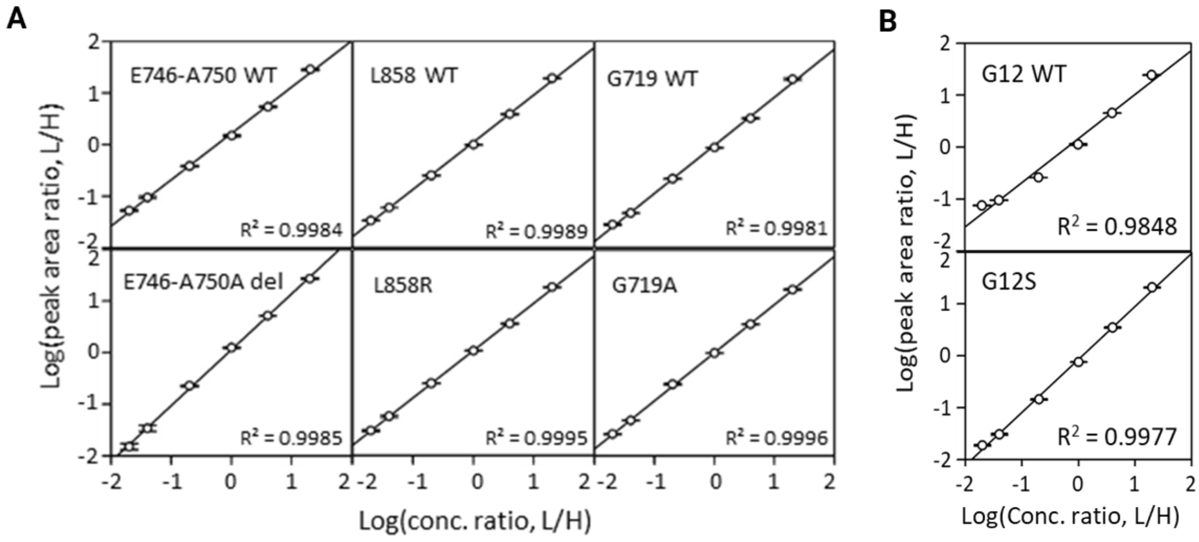

Figure S5. Quantification performance of EGFR and KRAS mutants and wilde types across dilution series

The calibration curves were constructed by 6 calibrators with serial dilutions of light peptides (0.5, 1, 5, 25, 100, and 500 fmol) and a fixed concentration of heavy peptides (25 fmol).

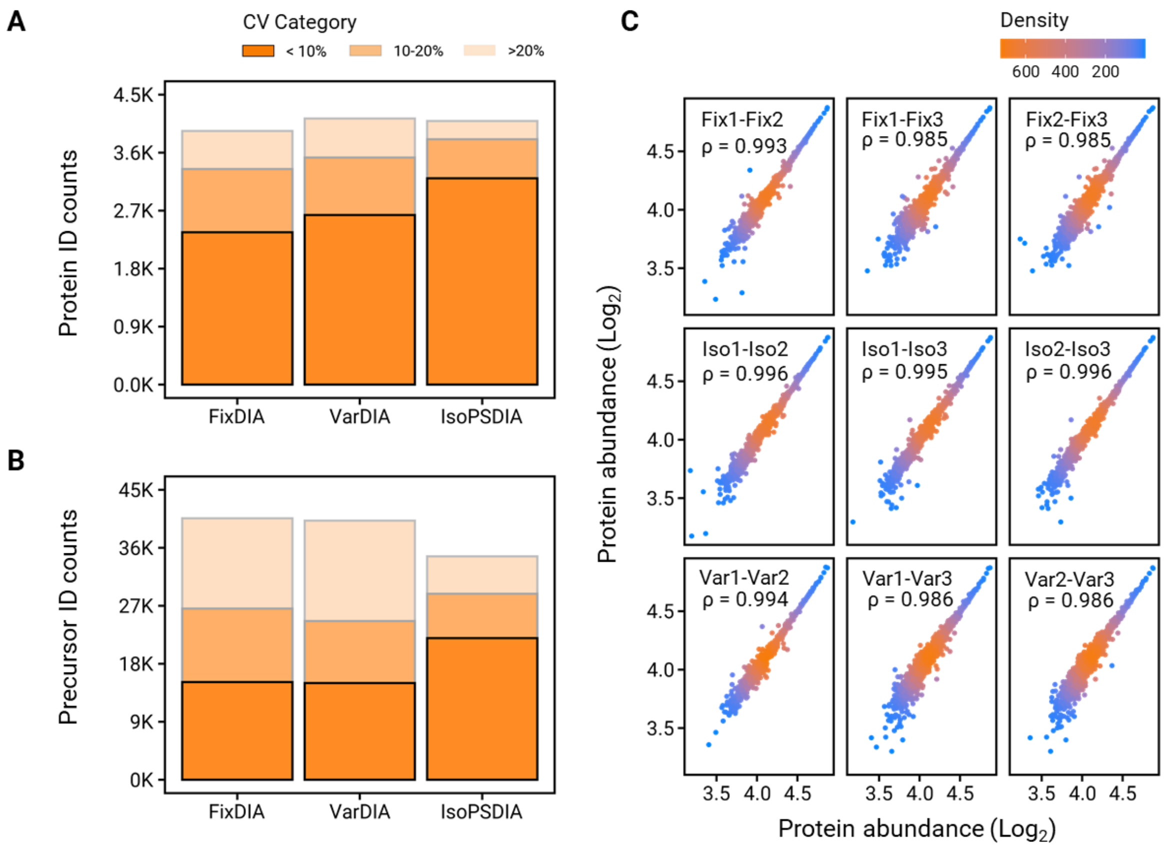

Figure S6. Comparison of proteome profiling coverage of PC9 between Fix-DIA, IsoPS-DIA, and Var-DIA methods. (A) Summary of protein identification numbers and (B) precursor identification numbers. (C) The relative abundance correlation analysis within each DIA method, showing spearman correlation rho values.

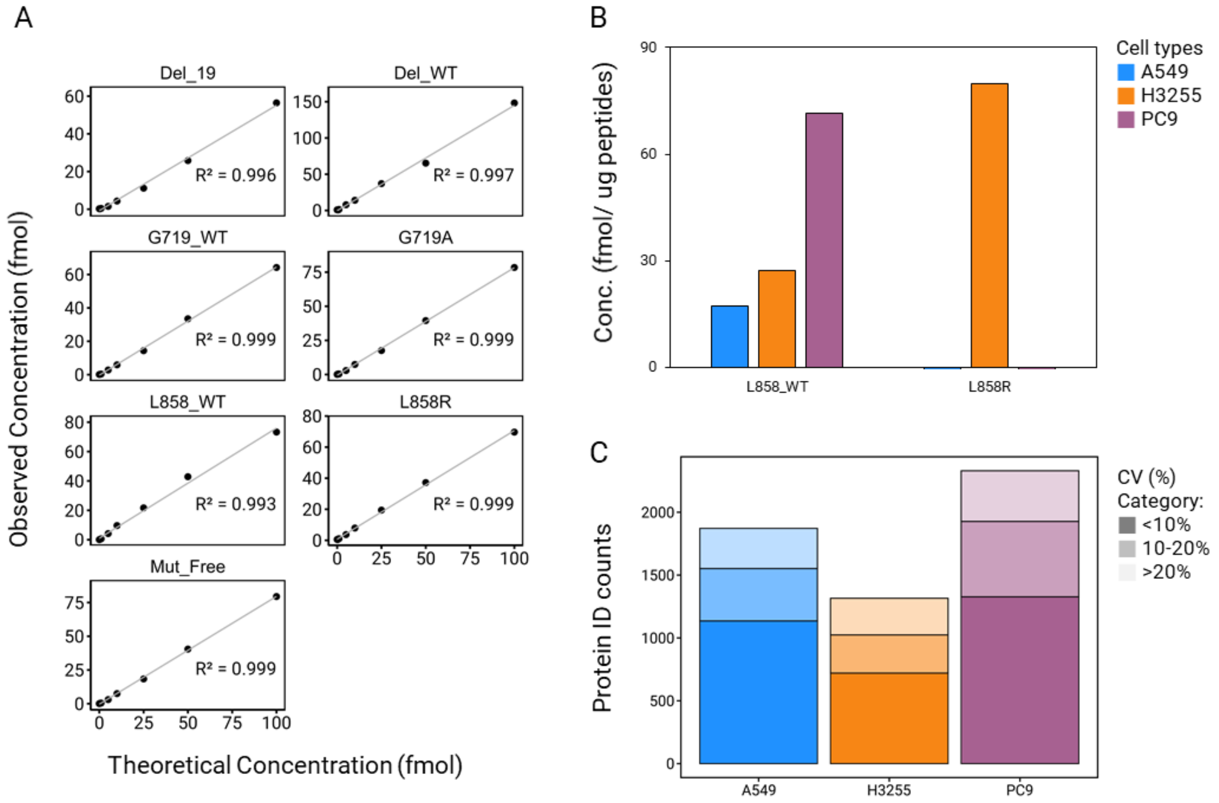

Figure S7. Targeted Quantitation and proteome profiling results in A549, H3255, and PC9 cell lines using IsoPS-DIA in Q-TOF mass spectrometer. (A) The calibration curves were constructed by 9 calibrators with serial dilutions of light peptides (0, 0.25, 0.5, 1, 5, 10, 25, 50, 100 fmol). (B) The absolute quantification results of L858R and corresponding wild-type peptides in the three cell lines. (C) The protein identification numbers with CV (%) count of the three cell lines.
